# Resource requirements for ecosystem conservation: A combined industrial and natural ecology approach to quantifying natural capital use in nature

**DOI:** 10.1002/ece3.9132

**Published:** 2022-07-31

**Authors:** Adam R. Mason, Alfred Gathorne‐Hardy, Chris White, Yves Plancherel, Jem Woods, Rupert J. Myers

**Affiliations:** ^1^ Department of Civil and Environmental Engineering Imperial College London London UK; ^2^ Global Academy of Agriculture and Food Security The University of Edinburgh Midlothian UK; ^3^ AECOM London UK; ^4^ Department of Earth Sciences and Engineering Imperial College London London UK; ^5^ Centre for Environmental Policy Imperial College London London UK

**Keywords:** biodiversity, conservation, natural resources, resource management

## Abstract

Socioeconomic demand for natural capital is causing catastrophic losses of biodiversity and ecosystem functionality, most notably in regions where socioeconomic‐and eco‐systems compete for natural capital, e.g., energy (animal or plant matter). However, a poor quantitative understanding of what natural capital is needed to support biodiversity in ecosystems, while at the same time satisfy human development needs—those associated with human development within socioeconomic systems—undermines our ability to sustainably manage global stocks of natural capital. Here we describe a novel concept and accompanying methodology (relating the adult body mass of terrestrial species to their requirements for land area, water, and energy) to quantify the natural capital needed to support terrestrial species within ecosystems, analogous to how natural capital use by humans is quantified in a socioeconomic context. We apply this methodology to quantify the amount of natural capital needed to support species observed using a specific surveyed site in Scotland. We find that the site can support a larger assemblage of species than those observed using the site; a primary aim of the rewilding project taking place there. This method conceptualises, for the first time, a comprehensive “dual‐system” approach: modelling natural capital use in socioeconomic‐and eco‐systems simultaneously. It can facilitate the management of natural capital at the global scale, and in both the conservation and creation (e.g., rewilding) of biodiversity within managed ecosystems, representing an advancement in determining what socioeconomic trade‐offs are needed to achieve contemporary conservation targets alongside ongoing human development.

## Graphical abstract

Schematic showing the changing ability to satisfy socioeconomic (human) and ecosystem well‐being needs concurrently, in response to four different scenarios (S1‐4) of socioeconomic development, corresponding to different distributions of natural capital stocks. SES is the ecosystem share; and SSE is the socioeconomic share.

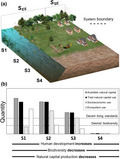

## 1 INTRODUCTION

The field of industrial ecology was developed to understand, analyze, and assess the environmental impacts of socioeconomic (human) actions (Graedel, 1996). It draws parallels between socioeconomic systems and ecosystems (Frosch, 1992) and argues that, given their inherent interdependence, the two must be studied simultaneously (Clift & Druckman, 2016; Graedel, 1996). This is particularly important when considering the provision and use of natural capital (stocks of natural resources such as fossil fuels, timber, and minerals [Mancini et al., 2017; Costanza & Daly, 1992; Goodland & Bank, 1995]), which act as a key interface between socioeconomic and ecosystems. Within industrial ecology, natural capital use is empirically modeled to systematically and quantitatively analyze the environmental impacts associated with human development (Weisz et al., 2015), and what effects human development will have on the quality and availability of natural capital in the future (Prescott‐Allen, 2001; Tilman, 1999). However, models of natural capital use are limited to socioeconomic analyses; they lack a holistic perspective and do not yet capture the natural capital demands of non‐human species.

At the most fundamental level, human development requires the use of natural capital to satisfy certain “human well‐being needs.” These are a set of essential (e.g., shelter, food, and water) and nonessential (e.g., access to technology) social, economic, and physiological requirements for good physical and mental health (Doyal & Gough, 1991; UN General Assembly, 1948). A robust understanding of natural capital use in socioeconomic systems allows it to be empirically modeled (Daniels & Moore, 2001). The associated environmental impacts can thereby be assessed (Bulle et al., 2019). Within the last two decades, a quantitative set of human well‐being needs has also been developed (Reid et al., 2005; Smith et al., 2013). This advancement has improved our ability to model and assess socioeconomic natural capital use associated with human development. It is now possible to infer the minimum level of natural capital use needed to support socioeconomic systems (Rao et al., 2019b), and assess the environmental impacts associated with achieving contemporary targets for human development—an important example being the sustainable development goals (SDGs) (United Nations Development Programme [UNDP], 2016).

All living organisms (plants, animals, fungi, etc.) demonstrate physiological needs. Like humans, they require water, energy, and nutrients, and in some cases—namely plant and animal species—they also demonstrate social needs (Poirier & Smith, 1974; Tedersoo et al., 2020). These requirements constitute the “well‐being needs” of individual plants and animals that make up ecosystems, analogous to human well‐being needs. However, a method to determine the well‐being needs of ecosystems has not yet been defined. This means that we do not know what natural capital is needed to support individual plants and animals, and by extension, biodiverse populations of species in ecosystems, nor do we know what natural capital must be allocated to ecosystems to protect essential ecosystem functions, i.e., the provision of natural capital, on which contemporary socioeconomic systems depend.

This knowledge gap is significant in the context of natural capital management: where socioeconomic‐ and ecosystems make use of the same, finite stocks of natural capital to satisfy their well‐being needs—namely land area, water, and energy (from food, etc.) (Andrews‐Speed et al., 2019; Ringler et al., 2013; Rugani et al., 2018)—the two systems must compete. Intuitively, the rate of natural capital production cannot be exceeded. Hence, where the combined rate of natural capital use across socioeconomic and ecosystems exceeds the rate of natural capital production, a trade‐off is necessary. This manifests as a compromise between satisfying human and ecosystem well‐being needs. Biased intentions to satisfy human well‐being needs in the present may therefore, inadvertently, limit our ability to satisfy those same human well‐being needs in the future, if ecosystems’ well‐being needs are sufficiently deprived (see Appendix 1) (Seddon et al., 2016).

### 1.1 Conceptual framework for quantifying ecosystem needs

Three variables are needed to assess the sustainability of natural capital use in socioeconomic systems: natural capital supply (stocks, rate of production), socioeconomic demand, and ecosystem demand. As ecosystem demand is not known, the tools we currently use to model natural capital use (e.g., material flow analyses [Brunner & Rechberger, 2005]) and assess the sustainability of its use (e.g., the ecological footprint [Wackernagel & Beyers, 2019]) are not yet comprehensive. By extension, current targets regarding sustainable human development may be flawed. For example, we can quantify what natural capital is needed to satisfy the socioeconomic SDGs (Rao et al., 2019b). However, we do not know what natural capital is needed to satisfy the ecological SDGs—those that relate to ecological conservation (namely goals 13, 14, and 15). This means that we are currently unable to determine whether the social, economic, and ecological SDGs can be achieved concurrently and whether enough natural capital remains once the socioeconomic SDGs are met to satisfy the ecological SDGs. Furthermore, we do not know whether the ecological SDGs, even if they were to be achieved in their entirety, adequately satisfy ecosystem well‐being needs underlying critical ecosystem functionality.

**FIGURE 1:**
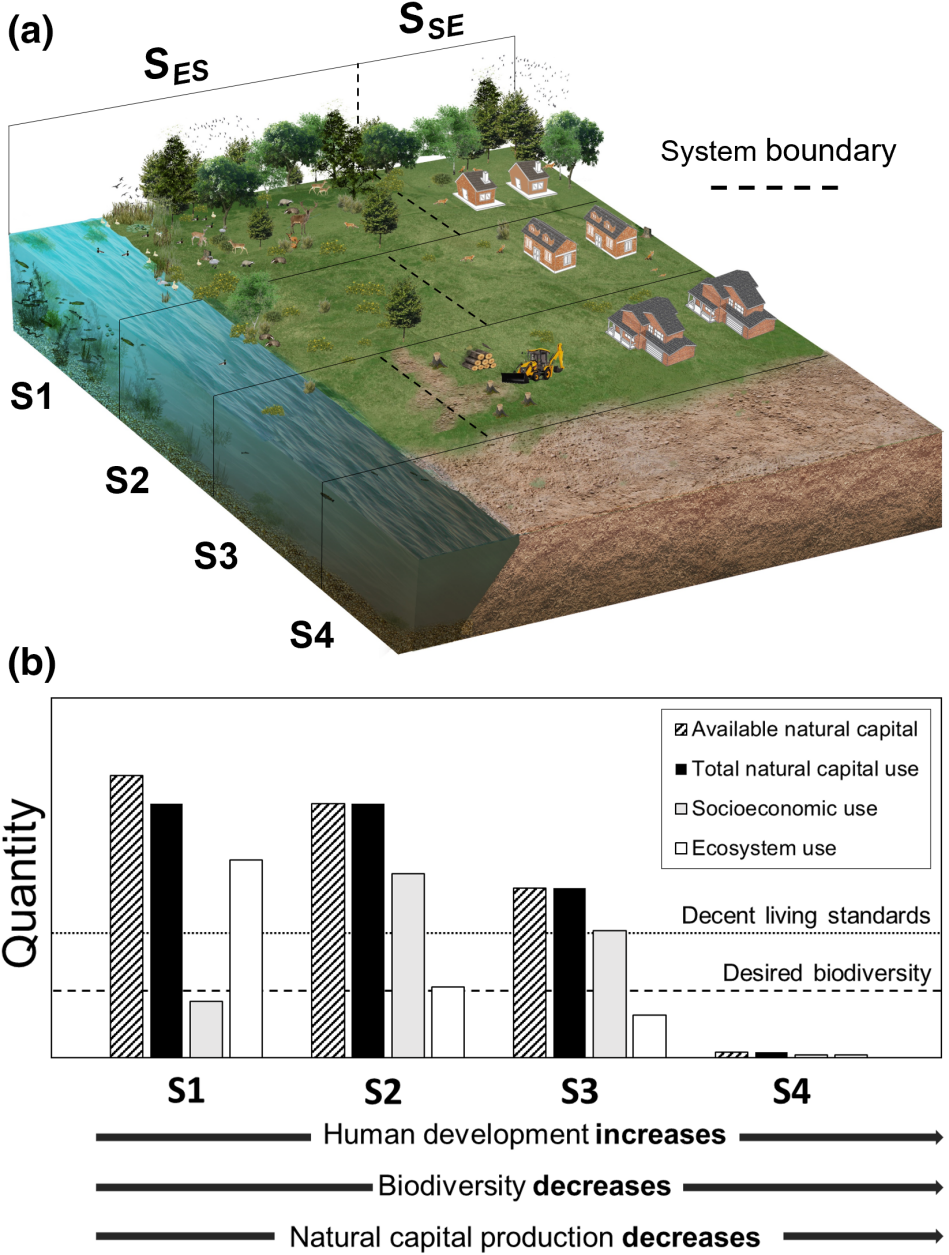
(a) Schematic showing the changing ability to satisfy socioeconomic‐ (human) and ecosystem well‐being needs concurrently, in response to four different scenarios of socioeconomic development, corresponding to different distributions of natural capital stocks. *S*_*ES*_ is the ecosystem share, and *S*_*SE*_ is the socioeconomic share. Scenario 1 (S1), high biodiversity and low human development, *S*_*ES*_ > *S*_*SE*_; Scenario 2 (S2), moderate biodiversity and moderate human development, *S*_*ES*_ < *S*_*SE*_; Scenario 3 (S3), low biodiversity and high human development, *S*_*ES*_ < < *S*_*SE*_; and Scenario 4 (S4), dual‐system collapse. (b) We present qualitatively the results of quantifying “ecosystem use” using our methodology and combining this with data describing “socioeconomic use” across the four scenarios. In (b), the dotted line labeled “decent living standards” indicates the point at which the socioeconomic population achieves decent living standards. The dashed line labeled “desired biodiversity” indicates the natural capital demand associated with a desired minimum ecosystem population. For illustrative purposes, “desired biodiversity” is here the minimum level of biodiversity needed to produce enough natural capital to sustain the socioeconomic population.

A comprehensive, systems approach to natural capital management is needed to determine what environmental conservation can be achieved alongside contemporary human‐development goals. Figure 1 illustrates how natural capital production, and human and ecosystem well‐being change in response to different distributions of (constant) natural capital stocks—here distributed between a socioeconomic system and an ecosystem—corresponding to four different scenarios of socioeconomic development (S1, Low human development, high ecosystem conservation; S2, moderate human development, moderate ecosystem conservation, S3, high human development, low ecosystem conservation; S4, collapse of the socioeconomic and ecosystem). Figure 1a shows increasing socioeconomic development (corresponding to increasing natural capital use) from S1 to S4, alongside decreasing biodiversity (as less natural capital is available for ecosystem use). In turn, decreasing biodiversity drives a reduction in natural capital production and hence its availability. As natural capital availability decreases, individuals in both the socioeconomic and ecosystem are less able to satisfy their well‐being needs (and vice versa). This deprivation results in the barren landscape illustrated in S4.

Figure 1b demonstrates qualitatively the application of our conceptual framework, showing the amount of natural capital allocated between the socioeconomic and ecosystems. Here, the natural capital allocated to the socioeconomic and ecosystems contribute to human development (e.g., to achieve a decent standard of living [Rao et al., 2019a]) and ecosystem conservation, respectively. This example also uses a constant socioeconomic population across S1‐S3. In Figure 1b, the four scenarios (S1‐S4) describe the following:

- S1: Low human development alongside high levels of biodiversity. A large amount of biodiversity is supported owing to abundant natural capital production, but insufficient human well‐being needs are met. Decent living standards are therefore not achieved across the socioeconomic population.
- S2: Human development increases, degrading the ecosystem. The provision of natural capital decreases. Much less biodiversity is supported, but this remains above the desired level. Sufficient human well‐being needs are now met; decent living standards are achieved across the socioeconomic population. This scenario of natural capital management represents a sustainable outcome, where human development and conservation targets are met simultaneously.
- S3: Human development and ecosystem degradation increase further. The provision of natural capital is further decreased. Sufficient human well‐being needs continue to be met; decent living standards are achieved across the socioeconomic population. However, the desired level of biodiversity can no longer be supported; there is insufficient natural capital to satisfy socioeconomic‐ and ecosystem well‐being needs concurrently.
- S4: A worst‐case scenario: Ecosystem needs continued to be deprived. The provision of natural capital is now insufficient to support either system. This scenario is unsustainable; socioeconomic‐ and ecosystem needs cannot be met, and the systems collapse.

S3 is a critical stage; it is a “tipping point,” where satisfying human well‐being needs diminishes our intergenerational needs (as illustrated in Figure A1, Appendix 1). Importantly, our ability to identify this tipping point in natural capital management is impaired by our current inability to quantify what natural capital is needed to support certain levels of biodiversity in ecosystems. As such, we cannot accurately distinguish between sustainable and unsustainable scenarios of natural capital management until both socioeconomic‐ and ecosystem demands are quantitatively described.

As the only species capable of safeguarding the biophysical environment, it is the responsibility of humans to consider the intergenerational needs of all species, not just our own. Comprehensive models of natural capital use, describing both socioeconomic‐ and ecosystem demand, are needed to assess and manage the trade‐offs triggered by human development and limited natural capital availability (Graedel, 1996). Especially where the impacts of socioeconomic use threaten to reduce production and hence the availability of natural capital. However, there is at present a fundamental knowledge gap that impairs our ability to develop such models. Specifically, data on what natural capital is needed to preserve and/or improve the life‐supporting functionality of ecosystems while achieving ongoing human development in socioeconomic systems.

### 1.2 Morphology–physiology relationships in the literature

Ecologists have long studied the relationship between morphology (e.g., body mass, body length, height) and physiology (e.g., metabolic rate, population density, water consumption) in animals and plants. These data are described extensively in the ecological literature and plethora of correlations between morphology and physiology have been demonstrated. Equations describing these correlations are termed “allometric equations” (Cyr & Pace, 1993). In his seminal work (McNab, 1963), McNab demonstrated the relationship between body mass and home range in mammals, suggesting that diet and metabolic rate are also important factors in an individual's land area use. This was later confirmed by Damuth (DAMUTH, 1987), and expanded upon: Variations in physiology are related to the trophic level and body mass ranges (Jetz et al., 2004; McNab, 2009; Meresman & Ribak, 2017; Nagy & Peterson, 1988; Silva & Downing, 1995) too. McNab's limited scope has been expanded beyond mammals, to include birds, reptiles, and insects (to a lesser extent), with allometric equations for plants (e.g., water uptake, energetics) also described (Biondini, 2008; Enquist et al., 1998).

Alternative means of predicting the land area use, metabolic requirements, and water consumption of species exist. These include species‐area curves (Scheiner, 2003; Schweiger et al., 2012), which are used to predict biodiversity (number of species) losses due to land area use changes, and modeling software that predicts species' response to environmental pressures (Holbrook et al., 2017); and metabolic rate predictions using heartrate measurements (McPhee et al., 2003), and oxygen consumption (Clark et al., 2006). Alternative means of predicting water consumption appear limited to agricultural contexts. Furthermore, these take the form of single‐species mathematical approaches (Appuhamy et al., 2016; Sexson et al., 2012), ultimately equivalent to allometric expressions. While alternatives exist, allometric equations remain a prominent means of predicting species physiology given their simple development/application, and the abundance of prerequisite data.

Information on natural capital use or production can be inferred where allometric equations are applied in the ecological literature. However, their application appears limited to ecological and agricultural literature. Examples include predicting biomass production (e.g., roots, timber, vegetation) across different land‐cover types (Vahedi, 2016); predicting the physiologies of undocumented species (Packard et al., 2009); and for design (Petherick & Phillips, 2009) and husbandry in an agricultural context. With the adjustment, and used concurrently, allometric equations can be incorporated into empirical models dealing with natural capital management and urban development. Despite its inherent benefits, this transference has not been realized. To overcome this, we build upon existing work on allometric equations and develop a generalisable method to quantify the use of natural capital in ecosystems, analogous to established methods to quantify human well‐being needs (Rao et al., 2019b), quantifying the natural capital cost associated with environmental conservation concurrent to that necessitated by human development.

## 2 METHOD

### 2.1 Land area

We develop a new set of allometric equations that relate the adult body mass (*M*_*i*_, kg) of individual mammals, birds, reptiles, and insects, to their land area use (*L*_*i*_, km^2^ individual^−1^) at the species level. The development of these equations improves upon existing work in the literature; we aggregate data corresponding to existing allometric equations for land area use—e.g., those published by (Damuth, 1987; Silva & Downing, 1995; Stephens et al., 2019; Robinson & Redford, 1986), and supplement these data with average adult body mass and trophic level data. We also incorporate the substantial datasets TetraDensity and PanTHERIA (Jones et al., 2009; Santini et al., 2018), which describe the population density (*ρ*_*i*_, individuals km^−2^) of mammal and bird species. These datasets were not captured in the preceding allometric equations for land area use since they were compiled after their publication of those preceding equations. The full, supplemented data used are presented in Tables S1–S8.

The reciprocal of population density (i.e., *ρ*_*i*_^−1^, km^2^ individual^−1^) is equivalent to land area use (Jetz et al., 2004), where population density describes the land area use of an individual as part of a wider single‐species population (Stephens et al., 2019). It is important to capture land area use in this way because land area is not a single‐use resource in ecosystems, nor is it used by individuals in solitude (Holling, 2001). The concept of population density is therefore preferable to other measures of land area use, such as home range, which employ an individual‐species approach to quantifying land area use (Jetz et al., 2004).

The allometric equations for the land area proposed in this paper were developed through linear regression analysis. The “fitlm” linear regression model (MathWorks, 2022) in the MATLAB computing environment was used to produce linear equations describing the relationship, “log_10_(adult body mass, kg) versus log_10_(population density, individuals km^−2^),” for terrestrial mammals, birds, reptiles, and insects. A substantial dataset consisting of over 17,000 data points was used to perform these analyses, which we present alongside corresponding adult body mass data and trophic level data in Tables S1–S8.

**FIGURE 2:**
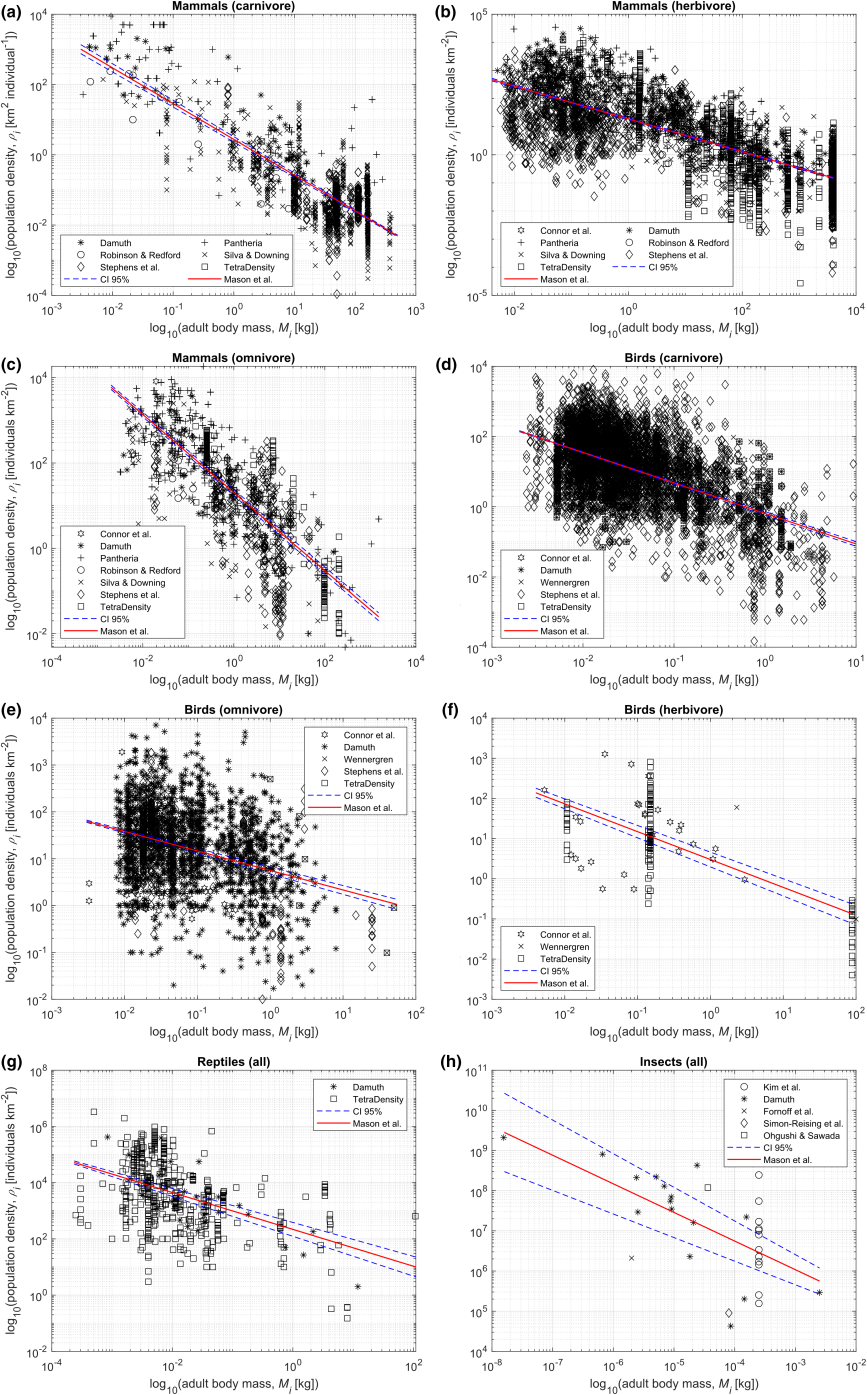
Plots showing the fits of our proposed allometric equations for land area use (red lines; see Table 1), and the 95% confidence intervals for each line of best fit, for mammals (a, carnivores; b, omnivores; c, herbivores); birds (d, carnivores; e, omnivores; f herbivores); (g) reptiles; and (h) insects. The sources of the data (markers) used are shown in the legends. In each case, the full dataset is available in the Data S1.

Previous studies demonstrate that mammal and bird species exhibit different adult body mass‐land area use relationships across the different trophic levels (Jenkins, 1981; Peters & Raelson, 1984). Therefore, we disaggregated population density data by trophic level (i.e., herbivore, omnivore, and carnivore) for mammals and birds, and performed regression analyses for each category case. Therefore, we disaggregated the population density data for mammal and bird species by trophic level. In contrast, the regression analyses for reptile and insect species were performed across all trophic levels. This is due to limited data at the species level in both cases. The results of the linear regression analyses are presented in Figure 2. The coefficients calculated for each linear regression are presented in Appendix 2.

In Figure 2, the equations describing each linear line of best fit (red lines) take the following form (Equation [1]):

(1) $${\mathrm{Log}}_{10}\left(\rho_{i}\right)=\left(b_{i}\pm e_{b,i}\right)\times {\mathrm{Log}}_{10}\left(M_{i}\right)+{\mathrm{Log}}_{10}\left(a_{i}\pm e_{a,i}\right),$$

where *ρ*_*i*_ (individuals km^−2^) is the population density of an individual (subscript *i*), *L*_*i*_ (km^2^ individual^−1^) is its reciprocal, the land area use of an individual; *M*_*i*_ (kg) is the adult body mass of the corresponding species; *a*_*i*_ and *b*_*i*_ are the intercept and the gradient (respectively) of the line of best fit—biological class‐specific parameters derived through regression analyses; and *e*_*a,i*_ *and e*_*b,i*_ are the standard error terms for *a*_*i*_ and *b*_*i*_, respectively*. a*_*i*_ *, b*_*i*_ *, e*_*a,i*_ *,* and *e*_*b,i*_ are presented in Table A2, Appendix 2.

The allometric equations for land area use (Equation [2]) are converted into the standard allometric equation form (Equation [5]) as follows:

(2) $${\mathrm{Log}}_{10}\left(\rho_{i}\right)={\mathrm{Log}}_{10}\left({M_{i}}^{b_{i}}\right)+{\mathrm{Log}}_{10}\left(a_{i}\right)$$

(3) $${\mathrm{Log}}_{10}\left(\rho_{i}\right)={\mathrm{Log}}_{10}\left(a_{i}\times {M_{i}}^{b_{i}}\right)$$

(4) $$\rho_{i}=a_{i}\times {M_{i}}^{b_{i}}$$

(5) $$L_{i}={\rho_{i}}^{-1}={\left(a_{i}\times {M_{i}}^{b_{i}}\right)}^{-1}$$

where *L*_*i*_ is the land area use of an individual (km^2^ individual^−1^), the reciprocal of that individual's population density (*ρ*_*i*_).

### 2.2 Water use

Terrestrial species either satisfy their water needs through active means, whereby water is ingested (i.e., drunk, or acquired through the diet); or through passive means, whereby water is absorbed directly from the atmosphere or produced metabolically (i.e., as a byproduct of respiration) (Nicholson, 2008; Schmidt‐Rohr, 2020). Here, “water use” is used to describe active water intake excluding that from the diet. This is because we are interested in the interaction between animal species and bodies of water that humans also utilize; metabolic water cannot be appropriated. We assume that dietary water requirements are satisfied when the energy requirements of an individual are met.

Ecological studies (e.g., [Calder, 1981]) have linked the water use of individual mammals and birds to their adult body masses. Allometric equations have subsequently been developed for wild and captive mammals (Equation [6]) and birds (Equation [6]):

(6) $$W_{i,\text{mammals}}=9.9\times 10^{-5}{\left(M_{i}\right)}^{0.9}$$

(7) $$W_{i,\text{birds}}=5.9\times 10^{-5}{\left(M_{i}\right)}^{0.67}$$

where *W*_*i, mammals*_ and *W*_*i, birds*_ (m^3^ individual^−1^ day^−1^) are terms quantifying the water use for an individual (subscript *i*) mammal and bird, respectively, and *M*_*i*_ (kg) is the adult body mass of the corresponding species. These equations have conventionally been used in an animal husbandry context to predict the water use needs of livestock (Ministry of environment & climate change strategy, 2001).

While the mechanism of drinking has been studied in some reptile species (e.g., [Cundall, 2000]), the drinking habits of insect and reptile species are not as well understood as those of mammals and birds (Nagy, 1982). Here we assume that insect species satisfy their need for water entirely through their diet, through metabolic means, and by absorbing water from the atmosphere (Barton‐Browne, 1964; Chapman et al., 2013; Nicholson, 2008). Similarly, we assume that reptiles satisfy their need for water entirely through their diets and through metabolic means (Martin & Sumida, 1997). Therefore, allometric equations describing water use by individual insects and reptiles are not presented here.

### 2.3 Energy

All living organisms require energy, in one form or another, to function. Terrestrial animal species are heterotrophic, they acquire energy from the food they eat (Nagy, 2005). Once eaten, this food is metabolized and used, for example, to regulate body temperature (McClune et al., 2015). The concept of “basal metabolic rate” (BMR), the rate at which energy is expended while an individual is at rest (McClune et al., 2015; Mcnab, 1997), is often used when describing the metabolism of animals (McClune et al., 2015). However, animals do not exist at rest. All animals must search for natural capital and/or interact with other individuals to satisfy their well‐being needs. A concept called the field metabolic rate (FMR) accounts for this additional exertion (Speakman, 1999). Therefore, we consider FMR to be a more appropriate concept for capturing energy use by terrestrial species. By describing the essential intake of food in terms of energy, this well‐being need can be compared across different taxonomic ranks and trophic levels (e.g., herbivore, carnivore, omnivore).

Allometric equations relating FMR to adult body mass for individual mammals, birds, and reptiles have been proposed with the general form shown in Equation (8) (Nagy et al., 1999):

(8) $$E_{i}=a_{i}\times {\left(M_{i}\right)}^{b_{i}}$$

where *E*_*i*_ (kJ individual^−1^ day^−1^) is the energy use of an individual (subscript *i*) mammal, bird, or reptile, *M*_*i*_ (kg) is the adult body mass of the corresponding species, and *a*_*i*_ and *b*_*i*_ are biological class‐specific parameters. Accounting for the inherent differences in metabolism across different trophic levels, we present allometric equations describing energy use for individual mammals (herbivores, Equation (9); omnivores, Equation (10); carnivores Equation (11)), birds (herbivores, Equation (12); omnivores, Equation (13); carnivores, Equation (14)), and reptiles (Equation [15]):

(9) $$E_{i,\text{mammals},\text{herbivore}}=688.4\times {\left(M_{i}\right)}^{0.646}$$

(10) $$E_{i,\text{mammals},\text{omnivore}}=652.1\times {\left(M_{i}\right)}^{0.678}$$

(11) $$E_{i,\text{mammals},\text{carnivore}}=791.2\times {\left(M_{i}\right)}^{0.85}$$

(12) $$E_{i,\text{birds},\text{herbivore}}=1,159.3\times {\left(M_{i}\right)}^{0.681}$$

(13) $$E_{i,\text{birds},\text{omnivore}}=716.6\times {\left(M_{i}\right)}^{0.628}$$

(14) $$E_{i,\text{birds},\text{carnivore}}=1264\times {\left(M_{i}\right)}^{0.705}$$

(15) $$E_{i,\text{reptiles}}=91.0\times {\left(M_{i}\right)}^{0.889}$$

An allometric equation describing the energy use of individual insects has previously been proposed by Ballesteros et al. (2018) (Ballesteros et al., 2018) (Equation [16]), which follows the same form as described in Equation (8).

(16) $$E_{i,\text{in}\sec\mathrm{ts}}=526\times {\left(M_{i}\right)}^{0.832}$$

However, Equation (16) describes energy use at the BMR, hence it does not describe the energy required to satisfy an individual's well‐being needs. Transformation of this equation to describe FMR is important to ensure its consistency with the equivalent allometric equations for mammals (Equations [(9), (10), (11)]), birds (Equations [(12), (13), (14), (15)]), and reptiles (Equation (15)). We transform Equation (18) based on the findings that *FMR* = 32*BMR, where *M*_*i, insects*_ < 1 × 10^−5^ kg; and *FMR* = 8*BMR, where *M*_*i, insects*_ ≥ 1 × 10^−5^ kg. This produces Equations ((17), (18)), which are now appropriate for quantifying the energy use associated with well‐being in individual insects.

(17) $$E_{i,\text{in}\sec\mathrm{ts}}=16,832{\left(M_{i}\right)}^{0.832}\;\text{where}\;M_{i}<1\times 10^{-5}\;\mathrm{kg}$$

(18) $$E_{i,\text{in}\sec\mathrm{ts}}=4,208{\left(M_{i}\right)}^{0.832}\;\text{where}\;M_{i}\geq 1\times 10^{-5}\;\mathrm{kg}$$

The assumptions that *FMR* = 8*BMR and *FMR* = 32*BMR are applied here as it has been shown that BMR scales with FMR for insects in a similar way to mammals and birds. For small mammals and birds, BMR is shown to scale to FMR according to *FMR* = 2*BMR (Golley, 1960). For insects, FMR is shown to be 32‐times greater than BMR in small insects (<1 × 10^−6^ kg), and 8‐times greater in large insects (≥1 × 10^−6^ kg; Niven & Scharlemann, 2005).

## 3 RESULTS

### 3.1 Proposed equations for quantifying ecosystem well‐being needs

Here, we compile a set of allometric equations (Tables 1 and 2) to quantify natural capital use in ecosystems, analogous to quantifying human well‐being needs in socioeconomic systems (Rao & Min, 2018). These equations relate the average adult body mass of individual terrestrial mammals, birds, reptiles, and insects to their land area use (*L*_*i*_, km^2^ individual^−1^), water use (*W*_*i*_, m^3^ individual^−1^ day^−1^), and energy use, i.e., from food or sunlight, (*E*_*i*_, kJ individual^−1^ day^−1^).

**TABLE 1:** Equations for land area use and energy use by individual mammals, birds, reptiles, and insects. Land area use is equal to the inverse of population density (i.e., *ρ* ^*−* *1*^), subscript *i* denotes an individual animal, and *M*_*i*_ (kg) is the average adult body mass of the corresponding species in each case.

Class	Trophic level	Land area use (km^2^ individual^−1^)	Energy use (kJ individual^−1^ day^−1^)
Mammals	Carnivore	${\left(2.74\times {\left(M_{i}\right)}^{-1.018}\right)}^{-1}$	$791.2\times {\left(M_{i}\right)}^{0.85}$
Mammals	Herbivore	${\left(18.84\times {\left(M_{i}\right)}^{-0.583}\right)}^{-1}$	$688.4\times {\left(M_{i}\right)}^{0.646}$
Mammals	Omnivore	${\left(20.28\times {\left(M_{i}\right)}^{-0.915}\right)}^{-1}$	$652.1\times {\left(M_{i}\right)}^{0.678}$
Birds	Carnivore	${\left(0.65\times {\left(M_{i}\right)}^{-0.868}\right)}^{-1}$	$1264\times {\left(M_{i}\right)}^{0.705}$
Birds	Herbivore	${\left(3.02\times {\left(M_{i}\right)}^{-0.693}\right)}^{-1}$	$1159.3\times {\left(M_{i}\right)}^{0.681}$
Birds	Omnivore	${\left(5.61\times {\left(M_{i}\right)}^{-0.416}\right)}^{-1}$	$716.6\times {\left(M_{i}\right)}^{0.628}$
Reptiles	All	${\left(218.3\times {\left(M_{i}\right)}^{-0.656}\right)}^{-1}$	$91.0\times {\left(M_{i}\right)}^{0.89}$
Insects	All	${\left(7852\times {\left(M_{i}\right)}^{-0.713}\right)}^{-1}$	$16,832\times {\left(M_{i}\right)}^{0.832}$ ^a^, $4208{\left(M_{i}\right)}^{0.832}$ ^b^

^a^ Where *M*_*i*_ < 1 × 10^−5^ kg.

^b^ Where *M*_*i*_ ≥ 1 × 10^−5^ kg.

**TABLE 2:** Equations for water use by individual mammals, birds, reptiles, and insects. The subscript *i* denotes an individual animal, and *M*_*i*_ (kg) is the average adult body mass of the corresponding species in each case.

Class	Water use (m^3^ individual^−1^ day^−1^)
Mammals	$9.9\times 10^{-5}\times {\left(M_{i}\right)}^{0.90}$
Birds	$5.9\times 10^{-5}\times {\left(M_{i}\right)}^{0.67}$
Reptiles	No data
Insects	No data

The allometric equations have the following general form:

(19) $$x_{i}=a_{i}\times {M_{i}}^{b_{i}}$$

where *x*_*i*_ is the natural capital use of an individual (subscript, *i*), e.g., km^2^ individual^−1^ for land area use (*x*_*i*_ = *L*_*i*_); *M*_*i*_ (kg) is the adult body mass of its corresponding species, and *a*_*i*_ and *b*_*i*_ are biological class‐specific parameters derived through regression analysis (see Table A2, Appendix 2). Our method uses the equations (in Tables 1 and 2), applied in conjunction with the results of ecological surveys (e.g., ecosystem surveys measuring the variables: Species name, number of individuals, location details, e.g., habitat type and climate; and where possible, body mass) to determine the land area, water, and energy needed to support desired individuals/networks (e.g., food webs) that comprise these surveyed ecosystems.

### 3.2 Interpreting land area use in multispecies populations

Land area use describes an activity that satisfies a range of essential (i.e., life‐supporting) physiological needs like thirst, hunger, and rest (e.g., space for nesting, and travel to nesting sites). It also describes other, less essential activities, for example, an individual animal's needs to socialize or stimulate themselves. In contrast, plants are sessile; their land area use is limited to essential needs, like the inception of sunlight and the uptake of water, nutrients, and gases associated with photosynthesis and respiration.

Different species co‐exist and make use of the same land area within ecosystems. Within a diverse population, social and physiological needs can be met through competitive and noncompetitive means. In the latter case, individuals of different species may satisfy their needs without compromising the ability of others to satisfy their own. For the former case, population density captures intraspecific (between the same species) but not interspecific (between different species) competition for land area. Inter‐species land area competition includes competition across different spatial planes (e.g., below ground, at ground level, in the canopy level, etc.), and across different trophic levels. While there have been some studies into the shared use of land area between sympatric species (e.g., [Avenant & Nel, 1997; Chamberlain & Leopold, 2005; Pigot et al., 2016; Steenhof & Kochert, 1985]), comprehensive data on interspecific competition is not readily available in the literature. However, we acknowledge that it is not reasonable to take the sum of individual land area use values to describe the land area needed to support a multispecies population.

**FIGURE 3:**
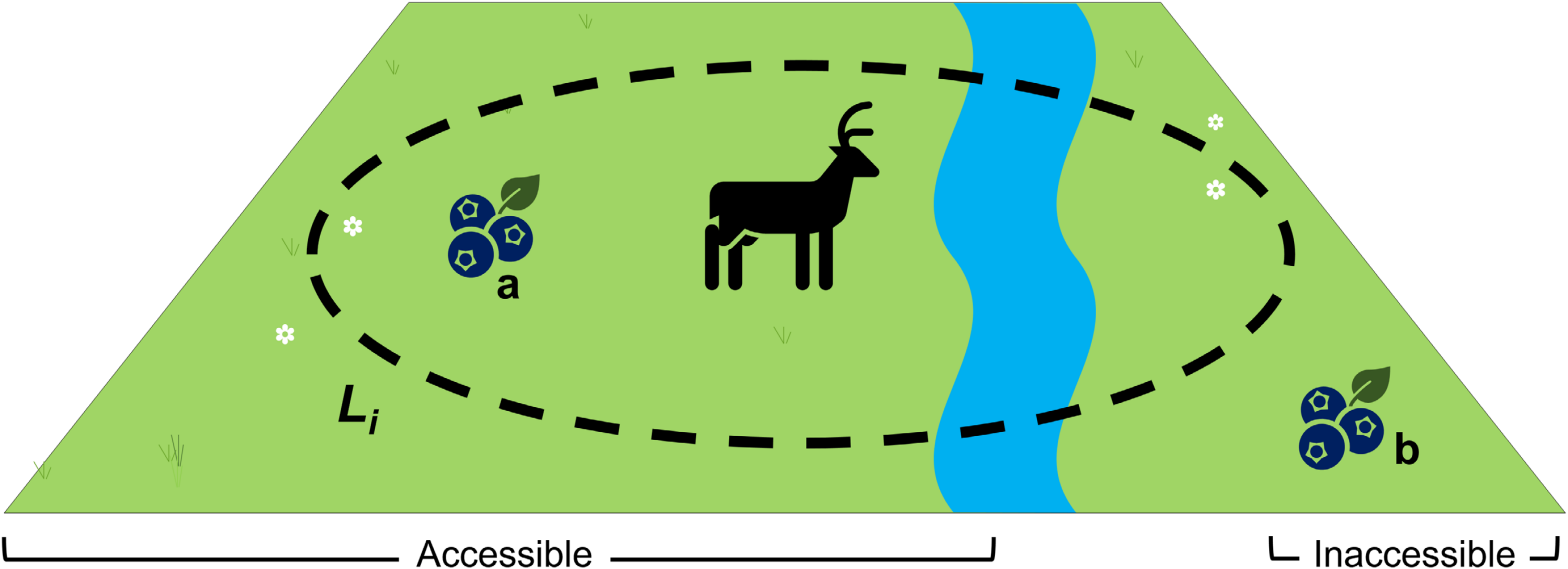
Illustration of “land area use” as a constraint in the estimation of ecosystem well‐being needs. *L*_*i*_ (dashed boundary) represents the land area use constraint; the land area which the individual animal (centre) is anticipated to use. The river acts as a physical barrier, making the right side inaccessible. The berries represent natural capital. The berries on the left (a) can be used by the individual (accessible and within the area *L*_*i*_), whereas the berries on the right (b) cannot (inaccessible and outside the area *L*_*i*_).

For these reasons, we take a conservative approach to interpreting land area use when applying our conceptual framework at this stage. We propose that land area use describes a constraint rather than a need in the same way “energy use” and “water use” do. Simply, the natural capital used by an individual should be located within the land area use range described by the land area use equation. Additionally, that natural capital must also be accessible to the individual, as illustrated in Figure 3, and of suitable quality. If natural capital is not available to an individual, we assume it cannot be used to satisfy that individual's well‐being needs. For illustrative purposes, the land area use by an individual is taken to describe a circular area in Figure 3.

An ecosystem's ability to support biodiversity depends on what energy is available there. Variations in what type and quality of energy are available across different climates and habitat types give rise to a global species richness gradient (Hawkins et al., 2003). The metabolic values calculated using the proposed equations for energy use (Table 1) make it possible to determine the land area needed to support herbivory within a multispecies population—the land area which supports primary producers, herbivores, and omnivores within an ecosystem—based on net primary productivity (NPP) data. This land area, which we call the “bioproductive land area, *L*_*BP*_” is calculated using Equation (20). Given the homogeneity of ecosystems, *L*_*BP*_ varies with climate, habitat type, and season. We apply and discuss Equation (20) in Section 3.3.2.

(20) $$L_{\mathrm{BP}}=\frac{E_{\text{population},\mathrm{BP}}}{C_{p}\times \eta_{i}\times \mathrm{NPP}\times s_{i}}$$

where *L*_*BP*_ (km^2^) is the bioproductive land area, *C*_*p*_ (kJ kg^−1^) is the average calorific value for plant matter within the bioproductive land area, *η*_*i*_ (kJ kJ^−1^) is the assimilation efficiency, i.e., calories extracted divided by calories available; *E*_*population,BP*_ (kJ population^−1^ day^−1^) is the total energy use of individual herbivores and omnivores satisfied through the consumption of primary producer species; *NPP* (kJ km^−2^ day^−1^) is the net primary productivity, and *s*_*i*_ (kg kg^−1^) is a coefficient that accounts for the suitability of biomass contributing to *NPP*. For example, if 10% of the NPP is used for herbivory (i.e., it is suitable and accessible), *s*_*i*_ = 0.1.

### 3.3 Applying the allometric equations

#### 3.3.1 Description of the surveyed site

**FIGURE 4:**
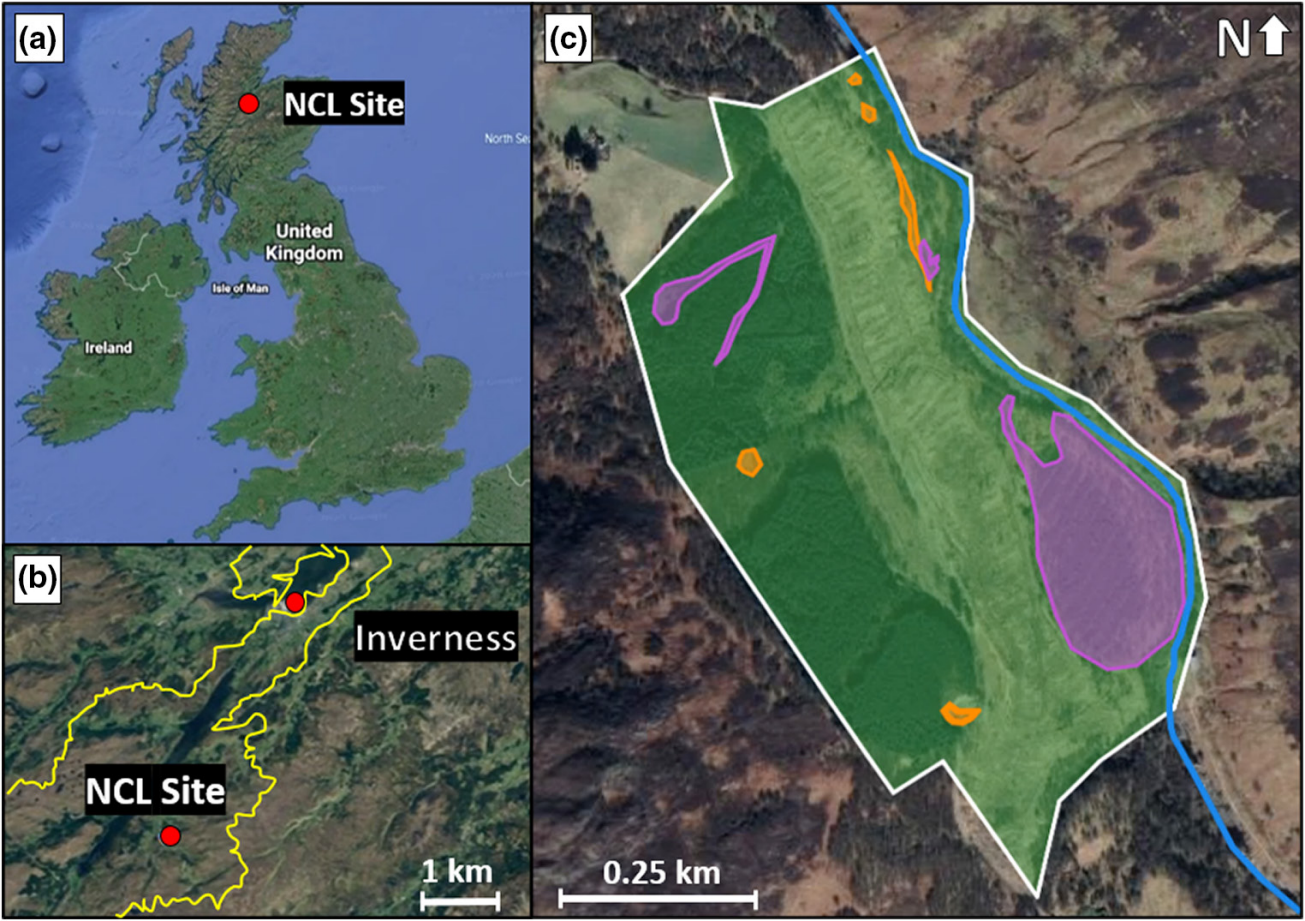
Location of the surveyed site within the UK (a) in proximity to Inverness, Scotland (b), and the habitat types at the NCL site (c). In (c), the white outline is the NCL site boundary; the green area is woodland; the orange area is seminatural grassland; the purple area is mountains, moorlands, and heath; and the blue area is freshwater, wetlands, and floodplains—the Fechlin River (Google, 2020a, 2020b, 2020c). The yellow line in (b) represents the catchment area of the River Ness. Map data were modified from Google Earth version 9.3.117.0: https://www.google.com/earth/.

We demonstrate the application of our allometric equations using a limited species survey from a site in the Scottish Highlands (Figure 4) that is being studied through a long‐term ecological survey (White, 2020). This site, formerly a conifer plantation, is the location for the National Capital Laboratory (NCL), a joint project between AECOM, “The Lifescape Project,” and the University of Cumbria (AECOM, 2020). The NCL was established in 2019 to re‐wild the site and monitor changes in biodiversity, habitat types, wildlife, and the quality of natural capital (e.g., soil and water quality) over a five‐year period. The aim of the project, in addition to the restoration and conservation of the site, is to understand the causal link between changes in biodiverse ecosystems, climate change, and biodiversity loss. The site itself has a total area of 0.426 km^2^ and includes the following broad habitat types: (1) woodland, (2) mountains, moorlands, and heath, (3) freshwater, wetlands, and floodplains, and (4) seminatural grassland.

The “freshwater, wetlands, and floodplains” habitat describes an on‐site river, which is not considered a terrestrial habitat and so is beyond the scope of this paper. This leaves an area of 0.403 km^2^, comprising three broad habitat types: woodland (84.7%); mountains, moors, and heaths (13.7%); and seminatural grassland (1.5%).

Through site surveys over a two‐month period, 45 species (192 individuals) were observed on the NCL site (White, 2020): 6 species of mammals (*N* = 9), 31 species of birds (*N* = 129), 7 species of insects (*N* = 52), and 1 species of reptile (*N* = 2) (Table S9). Once recorded, these species were each assigned an average adult body mass (Encyclopedia of Life, 2018). These adult body mass data are not included in the site surveys but are needed to apply the proposed allometric equations for land area use, energy use, and water use (Tables 1 & 2). We note that the number of insects observed in the NCL site is much lower than one would expect. The insect population does not exceed the number of vertebrates, which suggests that the NCL data do not accurately capture the abundance of species on the site. However, we view these NCL data as still being useful here to demonstrate the application of our allometric equations and as a basis for more comprehensive data collection in later stages of the site survey. Therefore, we treat the total land area use calculated using the NCL data as an underestimate of that used by biodiversity on site.

At this stage, our framework is applied only conceptually. It aims to describe what quantity of the natural capital produced at the NCL is currently used by biodiversity on site. In practice, these data would be used to understand the natural capital cost of supporting biodiversity at the NCL site. Specifically, to investigate scenarios of conservation/rewilding versus human development on site: to determine whether current levels of biodiversity (assuming the observed population = 1 unit of biodiversity) can be supported at the NCL site given its current natural capital stocks and coexisting socioeconomic use; and whether current or desired levels (*n* units) of biodiversity can be supported at the NCL site alongside increased human development (e.g., increased water abstraction, the development of infrastructure on site, etc). The effect of which is decreased natural capital production, and reduced access to natural capital stocks amongst animals (see Figure 1b). The application of our framework could therefore guide conservation and development practices at the NCL site, optimizing the site's ability to meet the ecosystem and socioeconomic needs simultaneously.

#### 3.3.2 Results and discussion

Tables 3 and 4 describe the natural capital requirements of the 45 species identified (the current, observed biodiversity) at the NCL site. These results do not describe the natural capital requirements associated with larger (i.e., minimum viable) populations of each species observed.

**TABLE 3:** Estimated land area use and energy use of species at the NCL site.

Class	Trophic level	*N*	Land area use (km^2^ population^−1^)	Energy use (kJ population^−1^ day^−1^)
Mammals	All	9	2.06	22,106
Mammals	Carnivore	1	0.48	989
Mammals	Omnivore	3	0.48	2713
Mammals	Herbivore	5	1.10	17,501
Birds	All	129	8.41	14,014
Birds	Carnivore	56	5.33	8214
Birds	Omnivore	66	2.93	5249
Birds	Herbivore	7	0.15	551
Reptiles	All	2	4.6 × 10^−4^	4
Insects	All	52	2.0 × 10^−5^	1035
Total	All	192	10.5	37,158

**TABLE 4:** Estimated water use of species at the NCL site

Class	Trophic level	*N*	Water use (m^3^ population^−1^ day^−1^)
Mammals	All	9	0.01
Birds	All	129	0.00
Reptiles	All	2	‐
Insects	All	52	‐
Total	All	192	0.01

The energy requirement for mammals is greatest despite the observed population of mammals being less than a tenth of the bird population. We attribute this result to the presence of sika deer at the NCL site, which has an average adult body mass of 42 kg. This is the greatest adult body mass of all the observed species at the NCL site, one that exceeds that which is possible for any flying bird species (O'Gorman & Hone, 2013). We suppose that this result may indicate a general trend that the energy requirement of mammals will be high, despite low mammalian populations relative to other species. Overall, these results indicate that birds and mammals will likely contribute most to the energy requirements associated with supporting biodiversity. Hence, the estimated energy use of mammals and birds may be sufficient to reliably describe that of a wider population when reptile and/or insect data are unavailable—a scenario, which reflects the current situation in the ecological literature.

The extent to which different species share the same land area is not known, so land area use is interpreted as a constraint. What can be identified from the NCL data (see Table S9) is that the greatest land area use at the NCL site is attributed to two species: *Meles meles* (0.48 km^2^) and *Cervus nippon* (0.47 km^2^). The NCL site itself is 0.43 km^2^, meaning all suitable natural capital within the NCL site boundaries area is accessible to both species. In fact, the species may make use of natural capital beyond the NCL site boundaries, to supplement their needs. All other individuals have land area use (constraint) values less than that of the NCL site.

Using our energy‐use values, we estimate the land area needed to satisfy the metabolic needs (energy use, Table 3) of primary consumers; we term this land area “bioproductive land area,” (*L*_*BP*_), and calculate it using Equation (20). Here we assume the NCL to be a temperate woodland (broadleaf, deciduous), with average “net primary productivity” (NPP, the rate at which organic compounds, in this class primary producers, are produced above ground, i.e., leaves, stems, fruit, etc.) of 918 kg km^−2^ day^−1^. A coefficient (*a*_*i*_) is applied to adjust the *NPP* because the quality of habitats at the NCL is considered poor (DeAngelis et al., 2013), and because some biomass will be of inadequate quality or incorrect type to support the observed species. In the absence of comprehensive data on the type of plant species present at the NCL site, or the dietary behavior of the observed species, we assume that 10% of *NPP* at the NCL site will be consumed by terrestrial species (Encyclopædia Britannica Inc., 2021). This effectively reduces the availability of energy in the bioproductive land area to 91.8 kg km^−2^ day^−1^. Finally, we also use an average calorific value for plant matter (*C*_*P*_, 18,812 kJ kg^−1^) (Yan et al., 2018) to describe the energy acquired from the bioproductive land area. This means that there is 1.73 × 10^7^ kJ km^−2^ day^−1^ of suitable plant matter produced at the NCL site.

We assume that all (100%) of herbivore energy use is satisfied through the consumption of primary producers and that seventy‐five percent (75%) of omnivore energy use is satisfied through the consumption of primary consumers. We acknowledge that a 75:25 ratio of meat to plant matter is unlikely across the observed omnivorous species. However, given the lack of data on the exact diets of the observed species, 75% is used here for the purposes of demonstrating this stage of the methodology. Using these assumptions, the total energy use that satisfied by the bioproductive land area (i.e., *E*_*Population,BP*_) is 27,051 kJ population^−1^ day^−1^ (see Table S10).

This value must next be adjusted to account for the (in)efficiency by which consumed material is converted to usable energy by an individual. Here we term this the “assimilation efficiency” (*η*_*i*_). The assimilation efficiency of herbivorous species ranges from 15 kJ kJ^−1^ to 80 kJ kJ^−1^ (Encyclopædia Britannica Inc., 2021). Again, owing to the absence of comprehensive data, an average assimilation efficiency of 47.5 kJ kJ^−1^ is used for the purpose of this calculation. This means that the herbivorous and omnivorous species in this example must in fact consume 5.69 × 10^4^ kJ population^−1^ day^−1^ to satisfy their metabolic needs.

Inputting the variables (*C*_*P*_, 18,812 kJ kg^−1^, *η*_*i*_ = 0.475, *NPP* = 918 kg km^−2^ day^−1^, *a*_*i*_ *=* 0.1 kg kg^−1^, *E*_*population,BP*_ = 27,051 kJ population^−1^ day^−1^) into Equation (20), we calculate that a bioproductive land area of 0.033 km^2^ population^−1^ is needed to support herbivory at the NCL site. This is equivalent to 8% of the NCL site (0.403 km^2^), suggesting herbivory may be adequately supported. The remaining metabolic needs would be met through carnivory, assuming adequate opportunity for predation exists beyond the boundaries of the NCL site.

In total, we calculate that 0.01 m^3^ day^−1^ water is required by the species observed on the NCL site. Given that the individual is not restricted to the NCL site, their water requirement can be satisfied using freshwater outside of the NCL site. It was estimated that 0.04 m^3^ day^−1^ freshwater was drawn from an on‐site well (for socioeconomic use) (White, 2020). This brings the total on‐site water demand to 0.05 m^3^ day^−1^. Although abstracted at different points (animals do not draw water from the well on site), the river Fechlin serves as a constant input of freshwater (one of many possible sources) to the site. Assuming the river Fechlin has a width of 15 m at the NCL site, 32,400 m^3^ [freshwater] m^−1^ [depth] day^−1^ [assuming an average velocity of 2160 m day^−1^ (Eltner et al., 2020)] traverses the NCL site. This suggests that freshwater is not the limiting resource at the NCL site. However, the upper limit of on‐site abstraction is difficult to predict. The river Fechlin is part of a larger catchment system, that of the River Ness (Scottish Environmental Protection Agency [SEPA], 2010), with a catchment area of 1849.1 km^2^ (Ness District Salmon Fishery Board and Scottish Fisheries Co‐ordination Centre, 2017; Scottish Environmental Protection Agency [SEPA], 2020) and hence many points of abstraction. Ultimately, combined water use (i.e., by socioeconomic and ecosystems) from rivers should generally be analyzed at the catchment system level to determine whether sufficient water is available to meet their combined needs.

Our findings suggest that conservation projects, like the NCL, would benefit from aligning their project boundaries with physical boundaries that delineate biodiverse ecosystems. The aims of the NCL project include the restoration and conservation of the site. However, because the observed species likely make use of the NCL as part of a wider land area, it is difficult to determine the full extent to which its restoration has on biodiversity. Thus, it may be difficult to distinguish which changes are due to the restoration of the NCL site, and which are due to changes beyond its boundaries. It would be beneficial to employ an ecological perspective to assess biodiversity change effectively, i.e., ecosystems should preferably be disaggregated along natural boundaries (i.e., physical boundaries, habitat type, and climate), rather than socioeconomic ones (i.e., political, and economic boundaries). For example, at the NCL site, the ecosystem perspective can be lost when socioeconomic boundaries are imposed. The results of its rewilding efforts might therefore be misinterpreted. The effects of its restoration would be better understood if the state of its surroundings were monitored simultaneously. Therefore, comprehensive ecological surveys of geographically isolated land areas, and applications of the allometric equations to those sites, present a promising next step to quantify land area requirements for biodiverse populations.

The NCL site comprises several habitat types (Figure 4): woodland (84.7%); mountains, moorlands, and heath (13.7%); and seminatural grassland (1.5%). These habitat types support biodiversity in different ways, meaning land area use at the class level will vary by habitat type. However, the distribution of species across the different habitat types at the NCL site is not reported, preventing analysis at this disaggregated level here. More information may be extracted by comparing biodiversity to disaggregated units of the site and its surroundings, like habitat type. The effects of increased land area use by socioeconomic systems could then be analyzed accounting for the importance of different habitat types for biodiversity amongst different species. This would help to identify which habitat types, and which stocks of natural capital, could be appropriated with the lowest impact on biodiversity. The ability to assess the possible effects of natural resource use on biodiversity would inform decision‐making on the allocation of land area (the type and the quantity) for conservation and for socioeconomic purposes. Such an improvement would facilitate the achievement of human‐development goals in ways that may prevent biodiversity loss.

At the fundamental level, all species require adequate space for their “well‐being.” The application of conceptual framework raises questions about how we interpret the needs of terrestrial species in a wider conservation context. For example, the Zoological Society of London (ZSL), the London Zoo, has a collection of over 600 species—over 19,000 individuals—at their Regent's Park site. However, this site has a land area of 0.15 km^2^, which is less than the land area estimated to be used by individual carnivorous mammals with body mass greater than 0.5 kg. This might suggest that biodiverse ecosystems can adapt to exist within greatly reduced land areas if trade‐offs between land area and nutritional requirements are managed. For example, through socioeconomic intervention such as the provision of food in these areas. This discrepancy might also suggest that we need to improve our understanding of what natural resources are needed to support individual species and, by extension, multispecies populations in biodiverse ecosystems.

## 4 OUTLOOK

Our proposed methodology offers a means by which the natural capital use in ecosystems may be quantified, analogous to human well‐being needs in socioeconomic systems. This advancement can facilitate the development of much‐needed comprehensive models of natural capital use, describing socioeconomic and ecosystems holistically. Critically, this will allow trade‐offs driven by the limited availability of natural capital—those between human and ecosystem well‐being, and subsequently those between human development and environmental conservation—to be identified, assessed, and managed. This will improve our ability to model and manage natural capital use at the global scale and facilitate the development of sustainable targets for achieving environmental conservation alongside human development.

Our work facilitates a more holistic approach to ecological conservation alongside human development. It addresses a fundamental knowledge gap across the ecological fields, to help satisfy one of the fundamental aims of industrial ecology: to study socioeconomic and ecosystems holistically. We are not calling for a conceptual shift from anthropocentrism to biocentrism. Instead, a pragmatic approach is needed; where new ecological data show human development causes excessive degradation of ecosystems, socioeconomic bias must give way to compromise by recognizing the trade‐offs in play.

We present an initial application of our methodology at this stage, based at the NCL site in the Scottish Highlands, which describes the application of our methodology in the context of rewilding (a form of ecosystem management). However, our proposed methodology can be applied in many other contexts. In the same way, the ecological footprint (Wackernagel & Beyers, 2019) describes the cost of global socioeconomic activities (number of planets), our methodology, in describing the natural capital cost of conserving biodiversity, provides an intuitive basis for natural capital management. Furthermore, by describing the natural capital cost associated with conservation analogous to that of satisfying human needs, there is potential to introduce our methodology to describe the cost of our conservation ambitions concurrent to socioeconomic activities. In a local context, our methodology can also complement assessments of urban metabolisms (e.g., [Hoekman & von Blottnitz, 2017]). These assessments use material flow analyses (MFAs), which describe the use of natural capital within defined boundaries—here a city. In a specific local context, our methodology can be incorporated into the life cycle assessment (LCA) framework (de Baan et al., 2013) to quantify the impact of urban development, for example, on local biodiversity—often called for in the literature (Winter et al., 2017).

Consider a plot of land within a city, for which several development plans are being considered. Each plan will require different quantities of materials, and each plan will alter the undeveloped plot in different ways (land area use change, the inclusion of blue‐green infrastructure, etc.). Given location‐specific data on urban animal populations (those expected to make use of the plot of land in question), we can describe the natural capital cost needed to support a population (1 unit) expected to use the plot of land. Hence, we can infer the number of populations (i.e., “*n*” units) each development scenario can support. By comparing these results, in conjunction with other LCA results, a more informed decision can be made on which development best suits the socioeconomic and conservational ambitions of the developers, the community, and so forth.

We suggest two priorities for the advancement of our methodology:

- Capturing the spatial and temporal heterogeneity of ecosystems (Libralato et al., 2006) within our framework. The use of natural capital in ecosystems varies at the local and global scale, in line with variables like habitat type, ecosystem structure, and the seasonality of plant species. Therefore, our allometric equations should be advanced to include additional terrestrial biological class, plant species, and marine species, while also disaggregating between different habitat types and climates.
- Quantifying the natural capital cost of conserving species critical to ecosystem functionality (pragmatic rather than comprehensive conservation, i.e., no loss of species [Wilson, 2016]). Thus, environmental‐conservation targets can be developed that protect critical ecosystem functionally with less socioeconomic compromise than a “no loss of species” approach. This application is contingent on sufficient data describing the role different species and intraspecific interactions play in critical ecosystem functionality.

Ultimately, however, our ability to understand the limits to ecologically sustainable human development is contingent on the collection and availability of ecological survey data across different habitat types and different biological classes, and at the global scale. In the absence of such data, the efficacy of contemporary human‐development and environmental‐conservation targets is uncertain—a problematic prospect given present‐day levels of ecological decline.

## AUTHOR CONTRIBUTIONS

**Adam R. Mason:** Conceptualization (equal); data curation (lead); formal analysis (lead); investigation (equal); methodology (equal); visualization (equal); writing – original draft (lead); writing – review and editing (lead). **Alfred Gathorne‐Hardy:** Methodology (supporting); visualization (supporting); writing – review and editing (equal). **Chris White:** Resources (supporting). **Yves Plancherel:** Writing – review and editing (supporting). **Jem Woods:** Conceptualization (supporting); writing – review and editing (supporting). **Rupert J. Myers:** Conceptualization (equal); methodology (equal); visualization (equal); writing – review and editing (equal).

## CONFLICT OF INTEREST

The authors declare no competing financial interests.

### OPEN RESEARCH BADGES

This article has earned an Open Data badge for making publicly available the digitally‐shareable data necessary to reproduce the reported results. The data is available at https://doi.org/10.5281/zenodo.6351417.

## Supporting information


**Appendix S1** Supporting Information

**Appendix S2** Supporting Information

## Data Availability

Data are available for this paper within a Supplementary Data file (https://doi.org/10.5281/zenodo.6351417). Correspondence and requests for materials should be addressed to a.mason19@imperial.ac.uk or r.myers@imperial.ac.uk.

## References

[ece39132-bib-0001] AECOM (2020). Natural Capital Laboratory. 2020. https://aecom.com/uk/natural‐capital‐laboratory/

[ece39132-bib-0002] Andrews‐Speed, P. , Zhang, S. , Andrews‐Speed, P. , & Zhang, S. (2019). The water‐energy‐food nexus. In China as a Global Clean Energy Champion (pp. 215–243). Springer Singapore. 10.1007/978-981-13-3492-4_9

[ece39132-bib-0003] Appuhamy, J. A. D. R. N. , Judy, J. V. , Kebreab, E. , & Kononoff, P. J. (2016). Prediction of drinking water intake by dairy cows. Journal of Dairy Science, 99(9), 7191–7205. 10.3168/jds.2016-10950 27320675

[ece39132-bib-0004] Avenant, N. L. , & Nel, J. A. J. (1997). Prey use by four syntopic carnivores in a strandveld ecosystem. African Journal of Wildlife Research, 27(3–4), 86–93.

[ece39132-bib-0005] Ballesteros, F. J. , Martinez, V. J. , Luque, B. , Lacasa, L. , Valor, E. , & Moya, A. (2018). On the thermodynamic origin of metabolic scaling. Scientific Reports, 8(1), 1448. 10.1038/s41598-018-19853-6 29362491 PMC5780499

[ece39132-bib-0006] Barton‐Browne, L. B. (1964). Water regulation in insects. Annual Review of Entomology, 9, 63–82. 10.1146/annurev.en.09.010164.000431

[ece39132-bib-0007] Biondini, M. (2008). Allometric scaling laws for water uptake by plant roots. Journal of Theoretical Biology, 251(1), 35–59. 10.1016/j.jtbi.2007.11.018 18171579

[ece39132-bib-0008] Brunner, P. H. , & Rechberger, H. (2005). Practical handbook of material flow analysis. Lewis Publishers.

[ece39132-bib-0009] Bulle, C. , Margni, M. , Patouillard, L. , Boulay, A. M. , Bourgault, G. , de Bruille, V. , Cao, V. , Hauschild, M. , Henderson, A. , Humbert, S. , Kashef‐Haghighi, S. , Kounina, A. , Laurent, A. , Levasseur, A. , Liard, G. , Rosenbaum, R. K. , Roy, P. O. , Shaked, S. , Fantke, P. , & Jolliet, O. (2019). IMPACT World+: A globally regionalized life cycle impact assessment method. International Journal of Life Cycle Assessment, 24(9), 1653–1674. 10.1007/s11367-019-01583-0

[ece39132-bib-0010] Calder, W. A. (1981). Scaling of physiological processes in Homeothermic animals. Annual Review of Physiology, 43, 301–322. 10.1146/annurev.ph.43.030181.001505 7011186

[ece39132-bib-0011] Chamberlain, M. J. , & Leopold, B. D. (2005). Overlap in space use among bobcats (Lynx Rufus), coyotes (Canis latrans) and gray foxes (Urocyon cinereoargenteus). American Midland Naturalist, 153(1), 171–179. 10.1674/0003-0031(2005)153[0171:OISUAB]2.0.CO;2

[ece39132-bib-0012] Chapman, R. F. , Simpson, S. J. & Douglas, A. E. (2013). *The insects: Structure and function*.P.929. http://search.ebscohost.com/login.aspx?direct=true&scope=site&db=nlebk&db=nlabk&AN=527855

[ece39132-bib-0013] Clark, T. D. , Butler, P. J. , & Frappell, P. B. (2006). Factors influencing the prediction of metabolic rate in a reptile. Functional Ecology, 20(1), 105–113. 10.1111/j.1365-2435.2006.01066.x

[ece39132-bib-0014] Clift, R. , & Druckman, A. (2016). Taking stock of industrial ecology. Springer International Publishin. 10.1007/978-3-319-20571-7

[ece39132-bib-0015] Costanza, R. , & Daly, H. E. (1992). Natural capital and sustainable development. Conservation Biology, 6(1), 37–46. https://www.jstor.org/stable/2385849

[ece39132-bib-0016] Cundall, D. (2000). Drinking in snakes: Kinematic cycling and water transport. Journal of Experimental Biology, 203(14), 2171–2185. 10862729 10.1242/jeb.203.14.2171

[ece39132-bib-0017] Cyr, H. , & Pace, M. L. (1993). Allometric theory: Extrapolations from individuals to communities. Ecology, 74(4), 1234–1245.

[ece39132-bib-0018] Damuth, J. (1987). Interspecific allometry of population density in mammals and other animals: The independence of body mass and population energy‐use. Biological Journal of the Linnean Society, 31(3), 193–246. 10.1111/j.1095-8312.1987.tb01990.x

[ece39132-bib-0019] Daniels, P. L. , & Moore, S. (2001). Approaches for quantifying the metabolism of physical economies: Part I: Methodological overview. Journal of Industrial Ecology, 5(4), 69–93. 10.1162/10881980160084042

[ece39132-bib-0020] de Baan, L. , Alkemade, R. , & Koellner, T. (2013). Land use impacts on biodiversity in LCA: A global approach. International Journal of Life Cycle Assessment, 18(6), 1216–1230. 10.1007/s11367-012-0412-0

[ece39132-bib-0021] DeAngelis, D. L. , Gardner, R. H. , & Shugard, H. H. (2013). NPP Multi‐Biome: Global IBP Woodlands Data, 1955‐1975, R1. ORNL DAAC. 10.3334/ORNLDAAC/198

[ece39132-bib-0022] Doyal, L. , & Gough, I. (1991). A theory of human need. Reprinted. Macmillan international higher education.

[ece39132-bib-0023] Eltner, A. , Sardemann, H. , & Grundmann, J. (2020). Technical Note: Flow velocity and discharge measurement in rivers using terrestrial and UAV imagery. Hydrology and Earth System Sciences, 24(3), 1429–1445. 10.5194/hess-2019-289

[ece39132-bib-0024] Encyclopædia Britannica Inc . (2021). Efficiency of solar energy utilization. (2021). https://www.britannica.com/science/biosphere/Efficiency‐of‐solar‐energy‐utilization#ref589416

[ece39132-bib-0025] Encyclopedia of Life . (2018). TraitBank. 2018. http://eol.org

[ece39132-bib-0026] Enquist, B. J. , Brown, J. H. , & West, G. B. (1998). Allometric scaling of plant energetics and population density. Nature, 395(6698), 163–165. 10.1038/25977

[ece39132-bib-0027] Frosch, R. A. (1992). Industrial ecology: A philosophical introduction. Proceedings of the National Academy of Sciences of the USA, 89, 800–803. 11607255 10.1073/pnas.89.3.800 PMC48328

[ece39132-bib-0028] Golley, F. B. (1960). Energy dynamics of a food chain of an old‐Field community. Ecological Monographs, 30(2), 187–206. https://www.jstor.org/stable/1948551

[ece39132-bib-0029] Goodland, R. , & Bank, T. W. (1995). The concept of environmental sustainability. Annual Review of Ecology and Systematics, 26, 1–24.

[ece39132-bib-0030] Google . (2020a). *Google Earth Version 9.3.117.0 ‐ Inverness, Scotland, co‐ordinates: 57°22′13"N 4°13′48"W*. 2020. https://earth.google.com/web/@57.31293638,‐4.3324268,218.76231676a,75248.68333787d,35y,0h,0t,0r

[ece39132-bib-0031] Google . (2020b). *Google Earth Version 9.3.117.0 ‐ NCL Site co‐ordinates 57°10′53”N 4°29′05”W, elevation 299 m*. 2020. https://earth.google.com/web/@57.18748723,‐4.48633425,268.16231138a,2502.38991116d,35y,‐0h,0t,0r

[ece39132-bib-0032] Google . (2020c). *Google Earth Version 9.3.117.0 ‐ United Kingdom, co‐ordinates: 50°22′59"N 2°52′14"E, elevation 1779 km*. 2020. https://earth.google.com/web/@54.39993743,‐4.37824067,336.81860678a,2035237.12481797d,35y,0h,0t,0r

[ece39132-bib-0033] Graedel, T. E. (1996). On the concept of industrial ecology. Annual Review of Energy and the Environment, 21, 69–98.

[ece39132-bib-0034] Hawkins, B. A. , Field, R. , Cornell, H. V. , Currie, D. J. , Ois, J.‐F. , Gan, G. , Kaufman, D. M. , Kerr, J. T. , Mittelbach, G. G. , Oberdorff, T. , O'brien, E. M. , Porter, E. E. , & Turner, J. R. G. (2003). Energy, water, and broad‐scale geographic patterns of species richness. Ecology, 84(12), 3105–3117.

[ece39132-bib-0035] Hoekman, P. , & von Blottnitz, H. (2017). Cape Town's metabolism: Insights from a material flow analysis. Journal of Industrial Ecology, 21(5), 1237–1249. 10.1111/jiec.12508

[ece39132-bib-0036] Holbrook, J. D. , Squires, J. R. , Olson, L. E. , Decesare, N. J. , & Lawrence, R. L. (2017). Understanding and predicting habitat for wildlife conservation: The case of Canada lynx at the range periphery. Ecosphere, 8(9), e01939. 10.1002/ecs2.1939

[ece39132-bib-0037] Holling, C. S. (2001). Understanding the complexity of economic, ecological, and social systems. Ecosystems, 4(5), 390–405. 10.1007/s10021-001-0101-5

[ece39132-bib-0038] Jenkins, S. H. (1981). Common patterns in home range‐body size relationships of birds and mammals. The American Naturalist, 118(1), 126–128.

[ece39132-bib-0039] Jetz, W. , Carbone, C. , Fulford, J. , & Brown, J. H. (2004). The scaling of animal space use. Science, 306(5694), 266–268. 10.1126/science.1102138 15472074

[ece39132-bib-0040] Jones, K. E. , Bielby, J. , Cardillo, M. , Fritz, S. A. , & O'Dell, J. (2009). PanTHERIA: A species‐level database of life history, ecology, and geography of extant and recently extinct mammals. Ecology, 90(9), 2648. 10.1890/08-1494.1

[ece39132-bib-0041] Libralato, S. , Christensen, V. , & Pauly, D. (2006). A method for identifying keystone species in food web models. Ecological Modelling, 195(3–4), 153–171. 10.1016/j.ecolmodel.2005.11.029

[ece39132-bib-0042] Mancini, M. S. , Galli, A. , Niccolucci, V. , Lin, D. , Hanscom, L. , Wackernagel, M. , Bastianoni, S. , & Marchettini, N. (2017). Stocks and flows of natural capital: Implications for ecological footprint. Ecological Indicators, 77, 123–128. 10.1016/j.ecolind.2017.01.033

[ece39132-bib-0043] Martin, K. L. M. , & Sumida, S. (1997). Chapter 12 ‐ water balance and the physiology of the amphibian to amniote transition. In Amniote origins: Completing the transition to land (p. 399). Elsevier.

[ece39132-bib-0044] MathWorks . (2022). Help Centre: fitlm. 2022. https://uk.mathworks.com/help/stats/fitlm.html;jsessionid=74b9eb14ad20dff99434d18954d9

[ece39132-bib-0045] McClune, D. W. , Kostka, B. , Delahay, R. J. , Montgomery, W. I. , Marks, N. J. , & Scantlebury, D. M. (2015). Winter is coming: Seasonal variation in resting metabolic rate of the European badger (Meles meles). PLoS One, 10(9), 1–17. 10.1371/journal.pone.0135920 PMC4564200 26352150

[ece39132-bib-0046] McNab, B. K. (1963). Bioenergetics and the determination of home range size. The American Naturalist, 97(894), 133–140. 10.1086/282264

[ece39132-bib-0047] Mcnab, B. K. (1997). On the utility of uniformity in the definition of basal rate of metabolism. Physiological Zoology, 70(6), 718–720. 10.1086/515881 9361146

[ece39132-bib-0048] McNab, B. K. (2009). Ecological factors affect the level and scaling of avian BMR. Comparative Biochemistry and Physiology ‐ A Molecular and Integrative Physiology, 152, 22–45. 10.1016/j.cbpa.2008.08.021 18805499

[ece39132-bib-0049] McPhee, J. M. , Rosen, D. A. S. , Andrews, R. D. , & Trites, A. W. (2003). Predicting metabolic rate from heart rate in juvenile Steller Sea lions Eumetopias jubatus. Journal of Experimental Biology, 206(11), 1941–1951. 10.1242/jeb.00369 12728015

[ece39132-bib-0050] Meresman, Y. , & Ribak, G. (2017). Allometry of wing twist and camber in a flower chafer during free flight: How do wing deformations scale with body size? Royal Society open Science, 4(10). 10.1098/rsos.171152 PMC5666286 29134103

[ece39132-bib-0051] Ministry of Environment & Climate Change Strategy . (2001). Animal weights and their food and water requirements .

[ece39132-bib-0052] Nagy, K. A. (1982). Field studies of water relations. Physiological Ecology, 12, 483–501.

[ece39132-bib-0053] Nagy, K. A. (2005). Field metabolic rate and body size. Journal of Experimental Biology, 208(9), 1621–1625. 10.1242/jeb.01553 15855393

[ece39132-bib-0054] Nagy, K. A. , Girard, I. A. , & Brown, T. K. (1999). Energetics of free‐ranging mammals, reptiles, and birds. Annual Review of Nutrition, 19, 247–277. 10.1146/annurev.nutr.19.1.247 10448524

[ece39132-bib-0055] Nagy, K. A. , & Peterson, C. C. (1988). Scaling of water flux rate in animals (2nd ed.). University of California Press.

[ece39132-bib-0056] Ness District Salmon Fishery Board and Scottish Fisheries Co‐ordination Centre . (2017). *Ness District Salmon Fishery Board: Ness System*. 2017. http://ness.dsfb.org.uk/ness‐system/

[ece39132-bib-0057] Nicholson, S. W. (2008). Index, W: Water balance. In J. L. Capinera (Ed.), Encyclopedia of entomology (2nd ed., p. 4153). Springer. 10.1007/978-1-4020-6359-6_2684

[ece39132-bib-0058] Niven, J. E. , & Scharlemann, J. P. W. (2005). Do insect metabolic rates at rest and during flight scale with body mass? Biology Letters, 1(3), 346–349. 10.1098/rsbl.2005.0311 17148203 PMC1617160

[ece39132-bib-0059] O'Gorman, E. J. , & Hone, D. (2013). Body size distribution of the dinosaurs. PLoS One, 7(12), 1–12. 10.1371/journal.pone.0051925 PMC3526529 23284818

[ece39132-bib-0060] Packard, G. C. , Boardman, T. J. , & Birchard, G. F. (2009). Allometric equations for predicting body mass of dinosaurs. Journal of Zoology, 279(1), 102–110. 10.1111/j.1469-7998.2009.00594.x

[ece39132-bib-0061] Peters, R. H. , & Raelson, J. V. (1984). Relations between individual size and mammalian population density. The American Naturalist, 124(4), 498–517.

[ece39132-bib-0062] Petherick, J. C. , & Phillips, C. J. C. (2009). Space allowances for confined livestock and their determination from allometric principles. Applied Animal Behaviour Science, 117(1–2), 1–12. 10.1016/j.applanim.2008.09.008

[ece39132-bib-0063] Pigot, A. L. , Tobias, J. A. , & Jetz, W. (2016). Energetic constraints on species coexistence in birds. PLoS Biology, 14(3), 1–21. 10.1371/journal.pbio.1002407 PMC4790906 26974194

[ece39132-bib-0064] Poirier, F. E. , & Smith, E. O. (1974). Socializing functions of primate play. American Zoologist, 14(1), 275–287. https://academic.oup.com/icb/article/14/1/275/2066761

[ece39132-bib-0065] Prescott‐Allen, R. (2001). The wellbeing of nations: A country‐by‐country index of quality of life and the environment (1st ed.). Island Press, In cooperation with International Development Research Centre, IUCN‐the World conservation union, International Institute for Environment and Development, food and agriculture Organization of the United Nations, Map Maker Ltd., UNEP World Co. 10.1002/9781118917046

[ece39132-bib-0066] Rao, N. D. , & Min, J. (2018). Decent living standards: Material prerequisites for human wellbeing. Social Indicators Research, 138, 225–244. 10.1007/s11205-017-1650-0 29950752 PMC6013539

[ece39132-bib-0067] Rao, N. D. , Min, J. , & Mastrucci, A. (2019a). Energy requirements for decent living in India, Brazil and South Africa. Nature Energy, 4(12), 1025–1032. 10.1038/s41560-019-0497-9

[ece39132-bib-0068] Rao, N. D. , Min, J. , & Mastrucci, A. (2019b). Energy requirements for decent living in India, Brazil and South Africa ‐ supplementary information. Nature Energy, 4(12), 1025–1032. 10.1038/s41560-019-0497-9

[ece39132-bib-0069] Reid, W. A. , Mooney, H. A. , Cropper, A. , Capistrano, D. , Carpenter, S. R. , Chopa, K. , Dasgupta, P. , Dietz, T. , Duraippah, A. K. , Hassan, R. , Kasperson, R. , Leemans, R. , May, R. M. , McMichael, T. & Pingali, P. (2005) Ecosystems and human well‐being: Synthesis. Millennium ecosystem assessment 10.5822/978-1-61091-484-0_1

[ece39132-bib-0070] Ringler, C. , Bhaduri, A. , & Lawford, R. (2013). The nexus across water, energy, land and food (WELF): Potential for improved resource use efficiency? Current Opinion in Environmental Sustainability, 5(6), 617–624. 10.1016/j.cosust.2013.11.002

[ece39132-bib-0071] Robinson, J. G. , & Redford, K. H. (1986). Body size, diet, and population density of Neotropical forest mammals. The American Naturalist, 128(5), 665–680.

[ece39132-bib-0072] Rugani, B. , Maes, J. , Othoniel, B. , Pulselli, F. M. , Schaubroeck, T. , & Ziv, G. (2018). Human‐nature nexuses: Broadening knowledge on integrated biosphere‐technosphere modelling to advance the assessment of ecosystem services. Ecosystem Services, 30, 193–199. 10.1016/j.ecoser.2018.04.002

[ece39132-bib-0073] Santini, L. , Isaac, N. J. B. , & Francesco, G. (2018). TetraDENSITY: A database of population density estimates in terrestrial vertebrates. Global Ecology and Biogeography, 27(7), 787–791. 10.1111/geb.12756

[ece39132-bib-0074] Scheiner, S. M. (2003). Six types of species‐area curves. Global Ecology and Biogeography, 12, 441–447.

[ece39132-bib-0075] Schmidt‐Rohr, K. (2020). Oxygen is the high‐energy molecule powering complex multicellular life: Fundamental corrections to traditional bioenergetics. ACS Omega, 5, 2221–2233. 10.1021/acsomega.9b03352 32064383 PMC7016920

[ece39132-bib-0076] Schweiger, O. , Betzholtz, P. , & Franze, M. (2012). Species‐area relationships are controlled by species traits species‐area relationships are controlled by species traits. PLoS One, 7(5), e37359. 10.1371/Citation 22629384 PMC3357413

[ece39132-bib-0077] Scottish Environmental Protection Agency (SEPA) . (2010). *North Highland area management plan catchment summaries: Ness*. (September).

[ece39132-bib-0078] Scottish Environmental Protection Agency (SEPA) . (2020). SEPA Water Level Data: River Ness. 2020. https://www2.sepa.org.uk/waterlevels/

[ece39132-bib-0079] Seddon, N. , Mace, G. M. , Naeem, S. , Tobias, J. A. , Pigot, A. L. , Cavanagh, R. , Mouillot, D. , Vause, J. , & Walpole, M. (2016). Biodiversity in the anthropocene: Prospects and policy. Proceedings of the Royal Society B: Biological Sciences, 283, 1–9. 10.1098/rspb.2016.2094 PMC5204156 27928040

[ece39132-bib-0080] Sexson, J. L. , Wagner, J. J. , Engle, T. E. , & Eickhoff, J. (2012). Predicting water intake by yearling feedlot steers 1,2. Journal of Animal Science, 90, 1920–1928. 10.2527/jas2011-4307 22205664

[ece39132-bib-0081] Silva, M. , & Downing, J. A. (1995). The allometric scaling of density and body mass: A nonlinear relationship for terrestrial mammals. The American Naturalist, 145(5), 704–727. 10.2307/j.ctvb6v6k6.16

[ece39132-bib-0082] Smith, L. M. , Case, J. L. , Smith, H. M. , Harwell, L. C. , & Summers, J. K. (2013). Relating ecoystem services to domains of human well‐being: Foundation for a U.S. index. Ecological Indicators, 28, 79–90. 10.1016/j.ecolind.2012.02.032

[ece39132-bib-0083] Speakman, J. R. (1999). The cost of living: Field metabolic rates of small mammals. Advances in Ecological Research, 30(C), 177–297. 10.1016/S0065-2504(08)60019-7

[ece39132-bib-0084] Steenhof, K. , & Kochert, M. N. (1985). Dietary shifts of sympatric buteos during a prey decline. Oecologia, 66, 6–16. 28310806 10.1007/BF00378546

[ece39132-bib-0085] Stephens, P. A. , Vieira, M. V. , Willis, S. G. , & Carbone, C. (2019). The limits to population density in birds and mammals. Ecology Letters, 22(4), 654–663. 10.1111/ele.13227 30724435 PMC6850427

[ece39132-bib-0086] Tedersoo, L. , Bahram, M. , & Zobel, M. (2020). How mycorrhizal associations drive plant population and community biology. Science, 367(6480), eaba1223. 10.1126/science.aba1223 32079744

[ece39132-bib-0087] Tilman, D. (1999). Diversity and production in European grasslands. Science, 286, 1099–1100.

[ece39132-bib-0088] UN General Assembly . (1948). Universal Declaration of Human Rights. UN General Assembly. 10.5195/rt.2019.591

[ece39132-bib-0089] United Nations Development Programme (UNDP) . (2016). Sustainable Development Goals. UNDP. https://www.undp.org/content/undp/en/home/sustainable‐development‐goals.html

[ece39132-bib-0090] Vahedi, A. A. (2016). Artificial neural network application in comparison with modeling allometric equations for predicting above‐ground biomass in the Hyrcanian mixed‐beech forests of Iran. Biomass and Bioenergy, 88, 66–76. 10.1016/j.biombioe.2016.03.020

[ece39132-bib-0091] Wackernagel, M. , & Beyers, B. (2019). Ecological Footprint. In Ecological footprint: Managing our biocapacity budget. Canada, New Society Publishers.

[ece39132-bib-0092] Weisz, H. , Suh, S. , & Graedel, T. E. (2015). Industrial ecology: The role of manufactured capital in sustainability. Proceedings of the National Academy of Sciences of the United States of America, 112(20), 6260–6264. 10.1073/pnas.1506532112 25986375 PMC4443377

[ece39132-bib-0093] White, C. X. (2020). Personal communication.

[ece39132-bib-0094] Wilson, E. O. (2016). Half‐earth: Our Planet's fight for life. W. W. Norton & Company.

[ece39132-bib-0095] Winter, L. , Lehmann, A. , Finogenova, N. , & Finkbeiner, M. (2017). Including biodiversity in life cycle assessment – State of the art, gaps and research needs. Environmental Impact Assessment Review, 67, 88–100. 10.1016/j.eiar.2017.08.006

[ece39132-bib-0096] Yan, P. , Xu, L. , & He, N. (2018). Variation in the calorific values of different plants organs in China. PLoS One, 13(6), e0199762. 10.1371/journal.pone.0199762 29953550 PMC6023129

