# Correction to “Resource requirements for ecosystem conservation: A combined industrial and natural ecology approach to quantifying natural capital use in nature”

**DOI:** 10.1002/ece3.10809

**Published:** 2024-06-04

Mason, A. J., Gathorne‐Hardy, A., White, C. D., Plancherel, Y., Woods, J., & Myers, R. J. (2022). *Ecology and Evolution*, 12(8). https://doi.org/10.1002/ece3.9132

Some data were incorrectly cataloged in Table S3 (herbivorous mammals) and Table S6 (herbivorous birds). Consequently, the corresponding equations in Table 1 were incorrect. The equations should have been:

Land use, herbivorous mammals: ${\left(55.91\times {\left(M_{i}\right)}^{-0.773}\right)}^{-1}$

Land use, herbivorous birds: ${\left(6.36\times {\left(M_{i}\right)}^{-0.438}\right)}^{-1}$

The following tables and figures have been updated accordingly:

- Table 1 and Figure 2 (main text), in which the equations are presented.
- Tables S3 and S6 (supplementary data) in which the underlying data are presented.
- Table 3 (main text) and Table S9 (supplementary data), in which the results applying the equations (the National Capital Laboratory case study) are presented.

The updated Table 1, Figure 2, and Table 3 are shown here. The updated Tables S3, S6, and S9 are shown in the updated Supplementary Data file, which is provided as online Supplementary Information to this article.

We apologize for this error.

**FIGURE 2:**
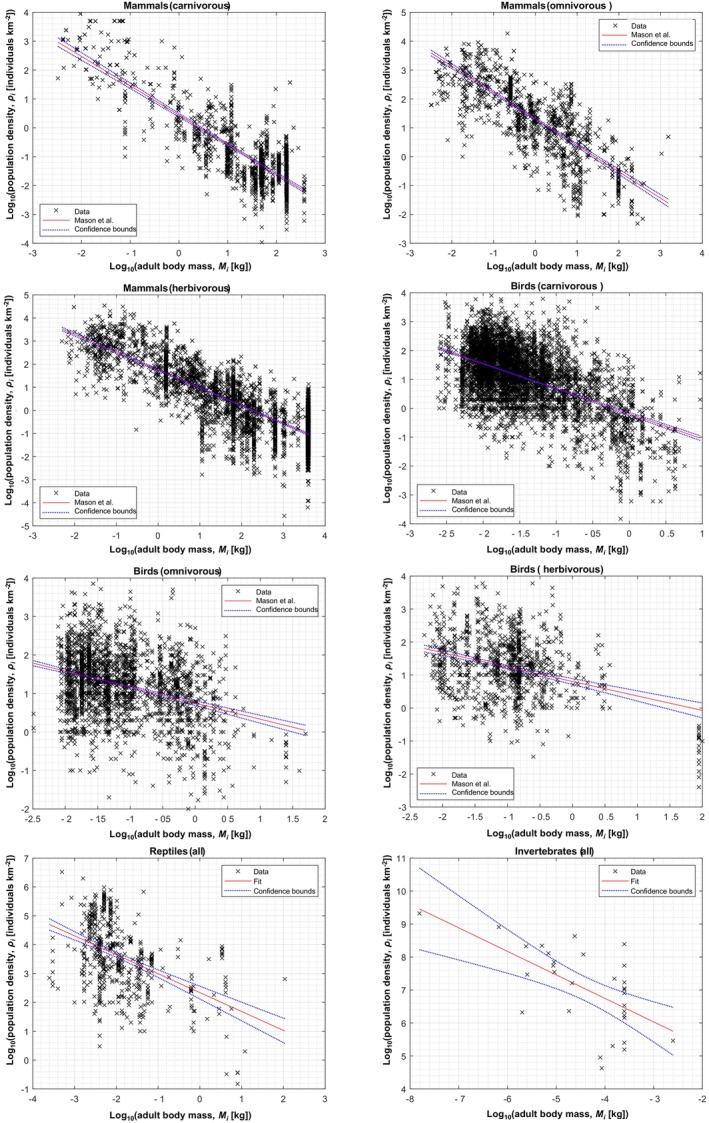
Plots showing the fits of our proposed allometric equations for land area use (red line, foreground; see Table 1), and the 95% confidence intervals for each line of best fit, for mammals (a, carnivores; b, omnivores; c, herbivores); birds (d, carnivores; e, omnivores; f, herbivores); g, reptiles; and h, insects. The sources of the data (markers, background) used are shown in the legends. In each case, the full dataset is available in the Supplementary Data document.

**TABLE 1:** Equations for land area use and energy use by individual mammals, birds, reptiles, and insects. Land area use is equal to the inverse of population density (i.e., ρ^−1^), subscript *i* denotes an individual animal, and *M*_*i*_ (kg) is the average adult body mass of the corresponding species in each case.

Class	Trophic level	Land area use (km^2^ individual^−1^)	Energy use (kJ individual^−1^ day^−1^)
Mammals	Carnivore	${\left(2.74\times {\left(M_{i}\right)}^{-1.018}\right)}^{-1}$	$791.2\times {\left(M_{i}\right)}^{0.85}$
Mammals	Herbivore	${\left(55.91\times {\left(M_{i}\right)}^{-0.773}\right)}^{-1}$	$688.4\times {\left(M_{i}\right)}^{0.646}$
Mammals	Omnivore	${\left(20.28\times {\left(M_{i}\right)}^{-0.915}\right)}^{-1}$	$652.1\times {\left(M_{i}\right)}^{0.678}$
Birds	Carnivore	${\left(0.65\times {\left(M_{i}\right)}^{-0.868}\right)}^{-1}$	$1264\times {\left(M_{i}\right)}^{0.705}$
Birds	Herbivore	${\left(6.36\times {\left(M_{i}\right)}^{-0.438}\right)}^{-1}$	$1159.3\times {\left(M_{i}\right)}^{0.681}$
Birds	Omnivore	${\left(5.61\times {\left(M_{i}\right)}^{-0.416}\right)}^{-1}$	$716.6\times {\left(M_{i}\right)}^{0.628}$
Reptiles	All	${\left(218.3\times {\left(M_{i}\right)}^{-0.656}\right)}^{-1}$	$91.0\times {\left(M_{i}\right)}^{0.89}$
Insects	All	${\left(7852\times {\left(M_{i}\right)}^{-0.713}\right)}^{-1}$	$16,832\times {\left(M_{i}\right)}^{0.832}$ ^a^, $4208{\left(M_{i}\right)}^{0.832}$ ^b^

^a^ Where *M*_*i*_ < 1 × 10^−5^ kg.

^b^ Where *M*_*i*_ ≥ 1 × 10^−5^ kg.

**TABLE 3:** Estimated land area use and energy use of species at the NCL site.

Class	Trophic level	*N*	Land area use^a^ (km^2^ population^−1^)	Energy use (kJ population^−1^ day^−1^)
Mammals	All	9	2.06	22,106
Mammals	Carnivore	1	0.48	989
Mammals	Omnivore	3	0.48	2713
Mammals	Herbivore	5	0.70	17,501
Birds	All	129	8.41	14,014
Birds	Carnivore	56	5.33	8214
Birds	Omnivore	66	2.93	5249
Birds	Herbivore	7	0.19	551
Reptiles	All	2	4.6 × 10^−4^	4
Insects	All	52	2.0 × 10^−5^	1035
Total	All	192	10.1	37,158

## Supporting information


Data S1.

