# Supplementary material for: Resource requirements for ecosystem conservation: A combined industrial and natural ecology approach to quantifying natural capital use in nature
Source: Ecol Evol. 2022 Jul 31;12(8):e9132. doi: 10.1002/ece3.9132 (PMC9339762; doi:10.1002/ece3.9132)
Supplement: Supplementary file 1 — Appendix S1 Supporting Information [file ECE3-12-e9132-s002.docx]

**Appendix 1**

The negative feedback loop (Fig. A1) illustrates how the unfulfillment of ecosystem well-being needs contributes to the reduced availability of natural capital in ecosystems, given the role of interspecific interaction in the production of natural capital in ecosystems. While many causal links between socioeconomic activity and ecosystem degradation are identified, our current lack of understanding on ecosystem well-being needs can further undermine our ability to meet human well-being needs and achieve human development goals.


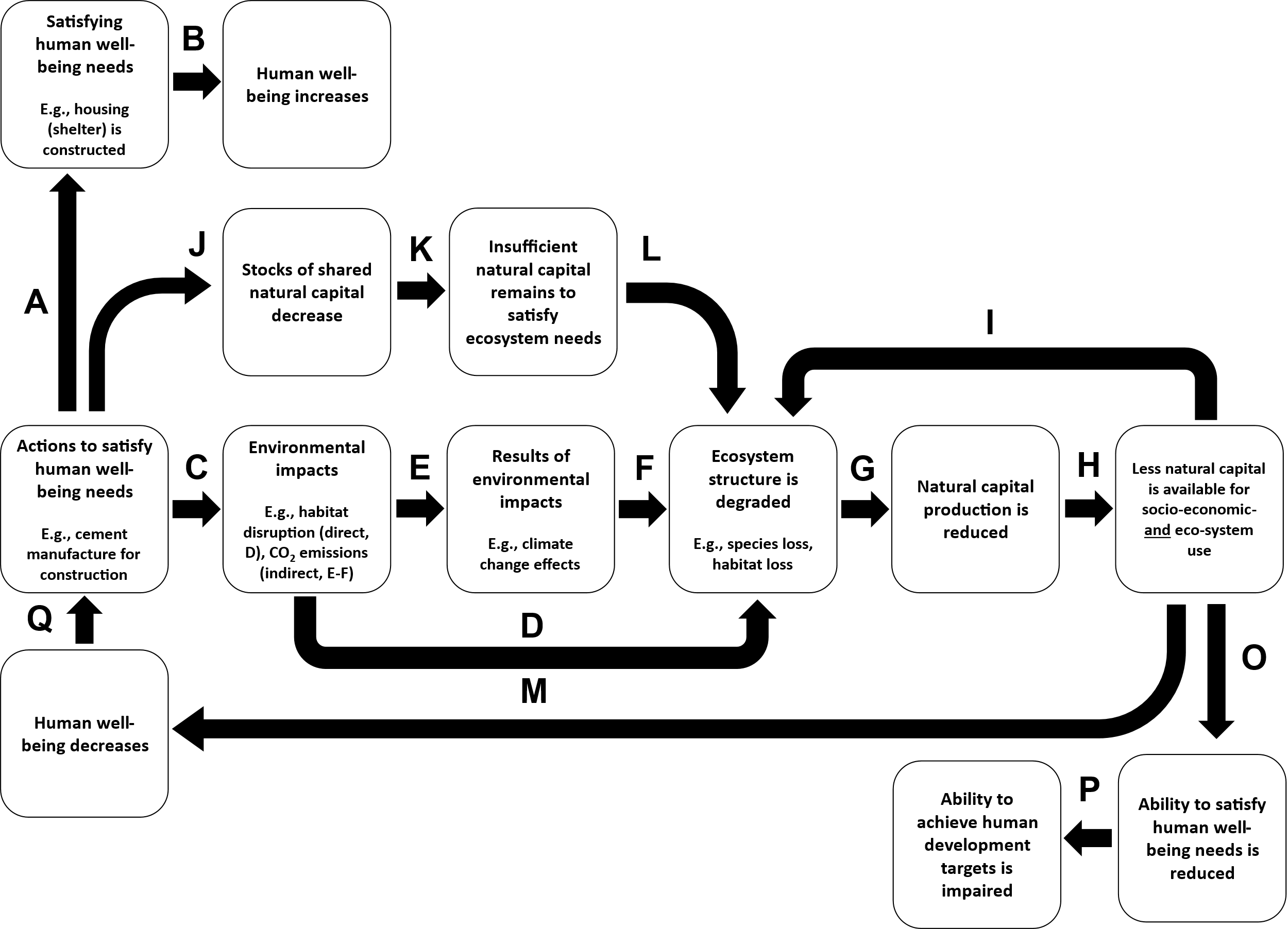


**Figure A1.** A ‘negative feedback’ scenario where ecosystem structure is degraded following two main causal pathways. First, due to the environmental impacts associated with meeting human well-being needs (C), either directly (D) or indirectly (E-F); and second, due to a trade-off between socio-economic and eco-systems driven by limited natural capital availability (J-L). This degradation reduces the production (G) and hence the availability (H) of natural capital, which further degrades ecosystem structure (I). This exacerbates the impacts of socio-economic activities. In response, human well-being decreases (M) and our ability to satisfy intergenerational human well-being needs is reduced (O). Our ability to achieve human development targets is thus impaired (P).

**Appendix 2**

Below we present the following regression coefficients derived from our regression analyses for log_10_(population density) versus log_10_(adult body mass)). These coefficients are all calculated using the ‘fitlm’ linear regression model (MathWorks, 2022) in the MATLAB computing environment.

**Table A1. Regression coefficients for log_10_(population density) versus log_10_(adult body mass) for terrestrial mammals, birds, reptiles, and insects.** Where *N* describes the number of observations, and *e* the root mean squared error.

| **Class** | **Trophic level** | ***N*** | ***e*** | ***R^2^*** | **p-value** |
| --- | --- | --- | --- | --- | --- |
|  |  |  |  |  |  |
| **Mammals** | Carnivore | 1323 | 0.76 | 0.666 | 1.46x10^-316^ |
| **Mammals** | Herbivore | 4296 | 0.964 | 0.547 | 0 |
| **Mammals** | Omnivore | 1458 | 0.796 | 0.611 | 8.64x10^-301^ |
| **Birds** | Carnivore | 6659 | 0.842 | 0.300 | 0 |
| **Birds** | Herbivore | 173 | 0.756 | 0.465 | 5.59x10^-25^ |
| **Birds** | Omnivore | 2521 | 0.808 | 0.113 | 2.43x10^-67^ |
| **Reptiles** | All | 578 | 1.07 | 0.205 | 1.44x10^-30^ |
| **Insects** | All | 31 | 0.992 | 0.377 | 2.39x10^-4^ |

In each case, the p-value is low, indicating that the null hypothesis can be rejected. This confirms what is already known: there is a relationship log_10_(population density) versus log_10_(adult body mass) across the four biological classes we analysed. There is great variation in the *R^2^* values in Table A1. The equations for mammals capture a greater amount of variance than the equations for birds, reptiles, and insects. The remaining variation could be attributed to variations in the habitats in which the species – those described across the data – were observed. Variations in natural capital availability and the rate of its production in one location may vary considerably to those in locations with different climates, habitat qualities, and population dynamics for example. Moreover, this data describes living, autonomous beings – some variation must be attributed to individual behaviour and personal choice, both of which are difficult to describe in a general (i.e., species-level) sense. To improve the analyses in future, it would be useful to investigate the relationship log_10_(population density) versus log_10_(adult body mass) disaggregated by habitat-type. While this is not easily done using data from Damuth (1986) and similar, more recently published databases (e.g., TetraDensity) permit such disaggregation for some datapoints. However, even in the TetraDensity database, many datapoints lack a ‘habitat type’ value.

In Table A2, we present the biological class-specific coefficients (*a_i_* and *b_i_*) which are described in Eqs. (1-5). A shown in Eq. 1 (main text), *a_i_* corresponds to the intercept of the linear line of best fit in each plot in Figure 2 (main text, red lines); *b­_i_* corresponds to the gradient the line in each case; and *e_a,i_ and e_b,i_* are the standard error terms for *a_i_* and *b_i_* respectively. These coefficients are all calculated using ‘fitlm’ linear regression model (MathWorks, 2022) in the MATLAB computing environment.

**Table A2. Species-specific coefficients for land area use**.

| **Class** | **Trophic level** | ***a_i_*** | ***e_a,i_*** | ***b_i_*** | ***e_b,i_*** |
| --- | --- | --- | --- | --- | --- |
| **Mammals** | Carnivore | 2.74 | 1.08 | -1.018 | 0.020 |
| **Mammals** | Herbivore | 18.84 | 1.04 | -0.583 | 0.008 |
| **Mammals** | Omnivore | 20.28 | 1.05 | -0.915 | 0.019 |
| **Birds** | Carnivore | 0.65 | 1.06 | -0.868 | 0.016 |
| **Birds** | Herbivore | 3.02 | 1.17 | -0.693 | 0.057 |
| **Birds** | Omnivore | 5.61 | 1.07 | -0.416 | 0.023 |
| **Reptiles** | All | 218.3 | 1.30 | -0.656 | 0.054 |
| **Insects** | All | 7852 | 5.90 | -0.713 | 0.170 |

We acknowledge that sampling bias might exist in some of the data used to perform our regression analyses (notably in Figures 2a and 2d-h; see main text) but addressing this sampling bias is beyond our scope at this stage. The sampling bias observed is due to an asymmetric distribution of data within the sources used to compile the data in Tables S1-S8 (see Supplementary Data). Where there is a bias towards individuals of lower log_10_(adult body mass), e.g., Figures 2d-g, the equation for land area use (Table 1, main text) may be an underestimate. Conversely, where there is a bias towards individuals of higher log_10_(adult body mass), e.g., Figures 2a and 2h, the equation for land area use may be an overestimate. In each case, we consider the line of best fit to be sufficiently robust since the relationships between log_10_(population density) and log_10_(adult body mass) are generally comparable (see Appendix 3) to those established in earlier publications: e.g., those published by (Damuth 1987), (Silva & Downing 1995), (Stephen et al., 2019), (Robinson & Redford, 1986).

**Appendix 3**

In most cases, our proposed equations for the land area use of mammals possess slopes that lie within the ranges of those developed by Damuth (1987) and Silva & Downing (1995), whose equations possess slopes ranging from –0.73 to –0.96, and –0.25 to –1.31, respectively (Damuth, 1987; Silva & Downing, 1995). For mammals, the plotted lines have comparable slopes; the exception being the equations from Silva & Downing (1995) for omnivores with adult body mass >100 kg and adult body mass <0.1 kg (Fig. A2b). We attribute this to the fact that the authors disaggregate the mammalian population density data by body mass as well as trophic level. By reducing the dataset size, the authors were able to identify different relationships between adult body mass and population density (Currie et al., 1993). However, it is difficult to determine whether the reduced datasets used were sufficiently large enough to produce accurate allometric equations for population density that may be applied with confidence at the biological class-level.


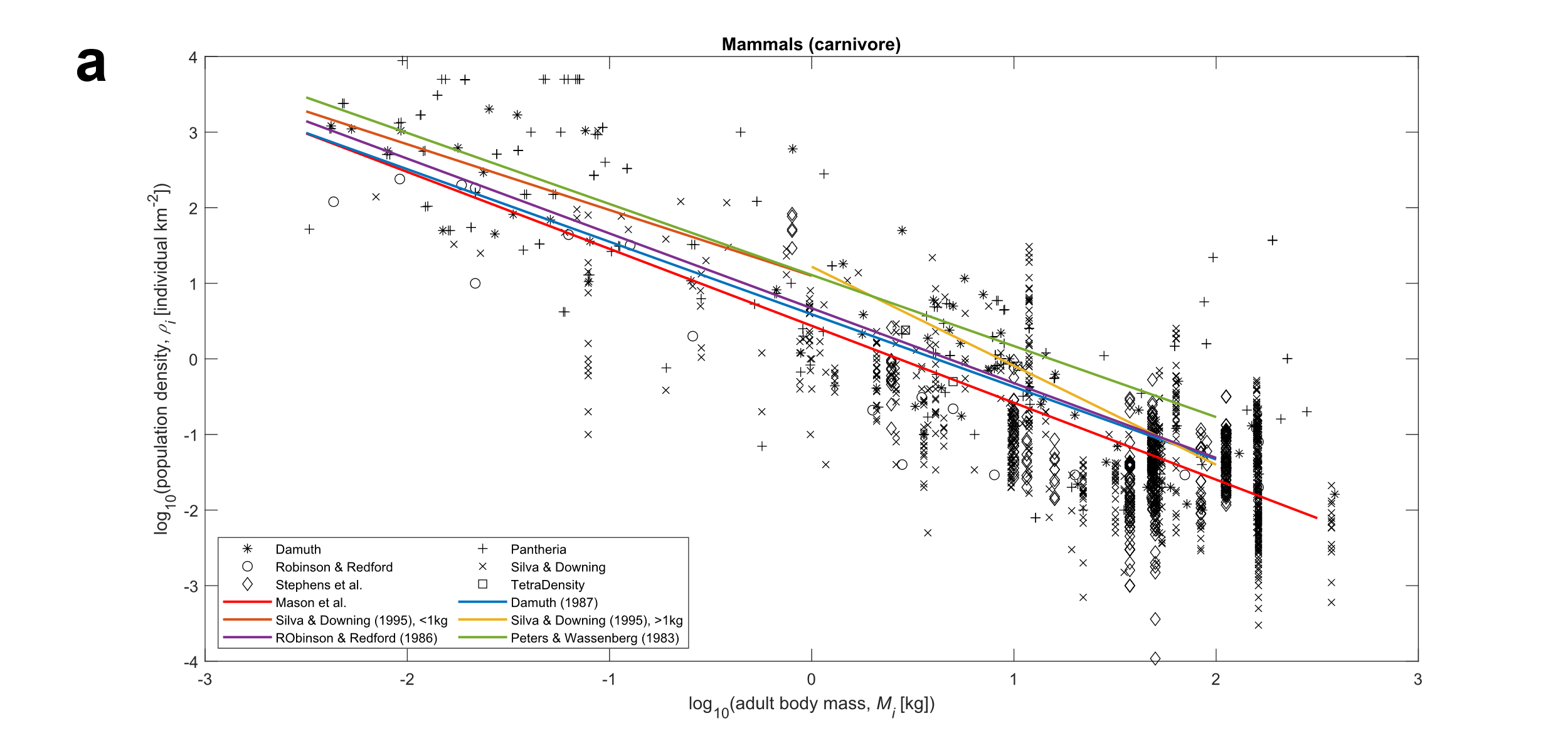


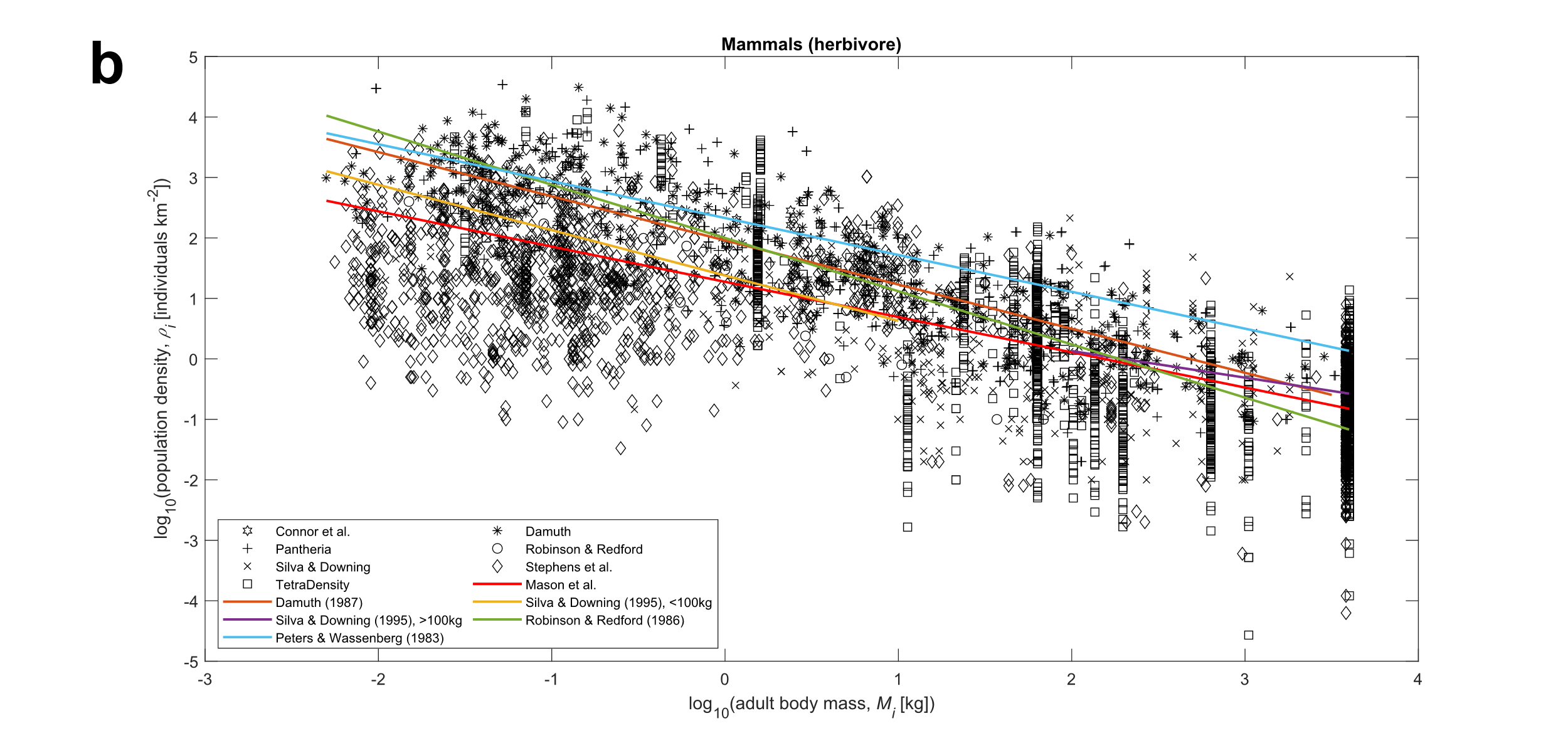


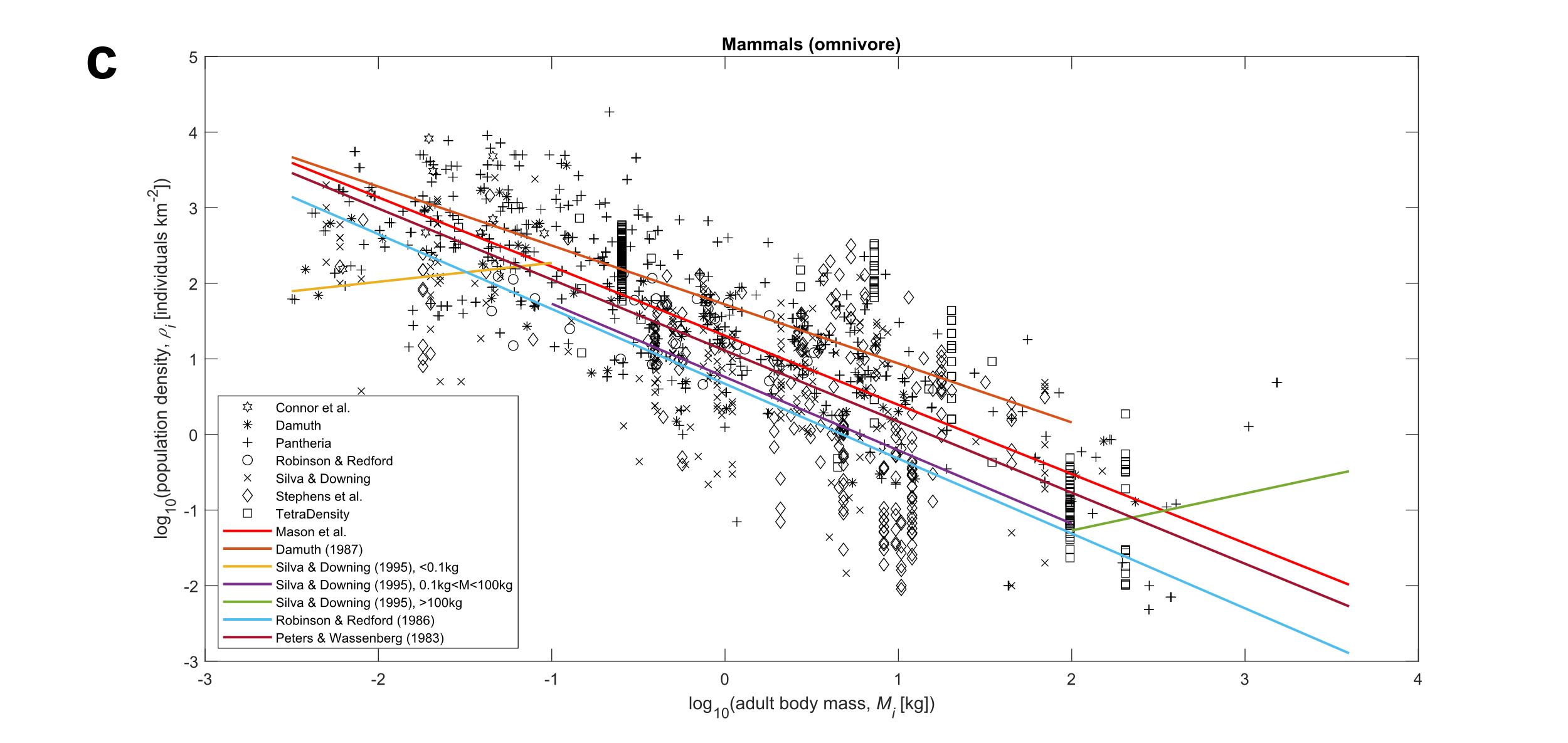


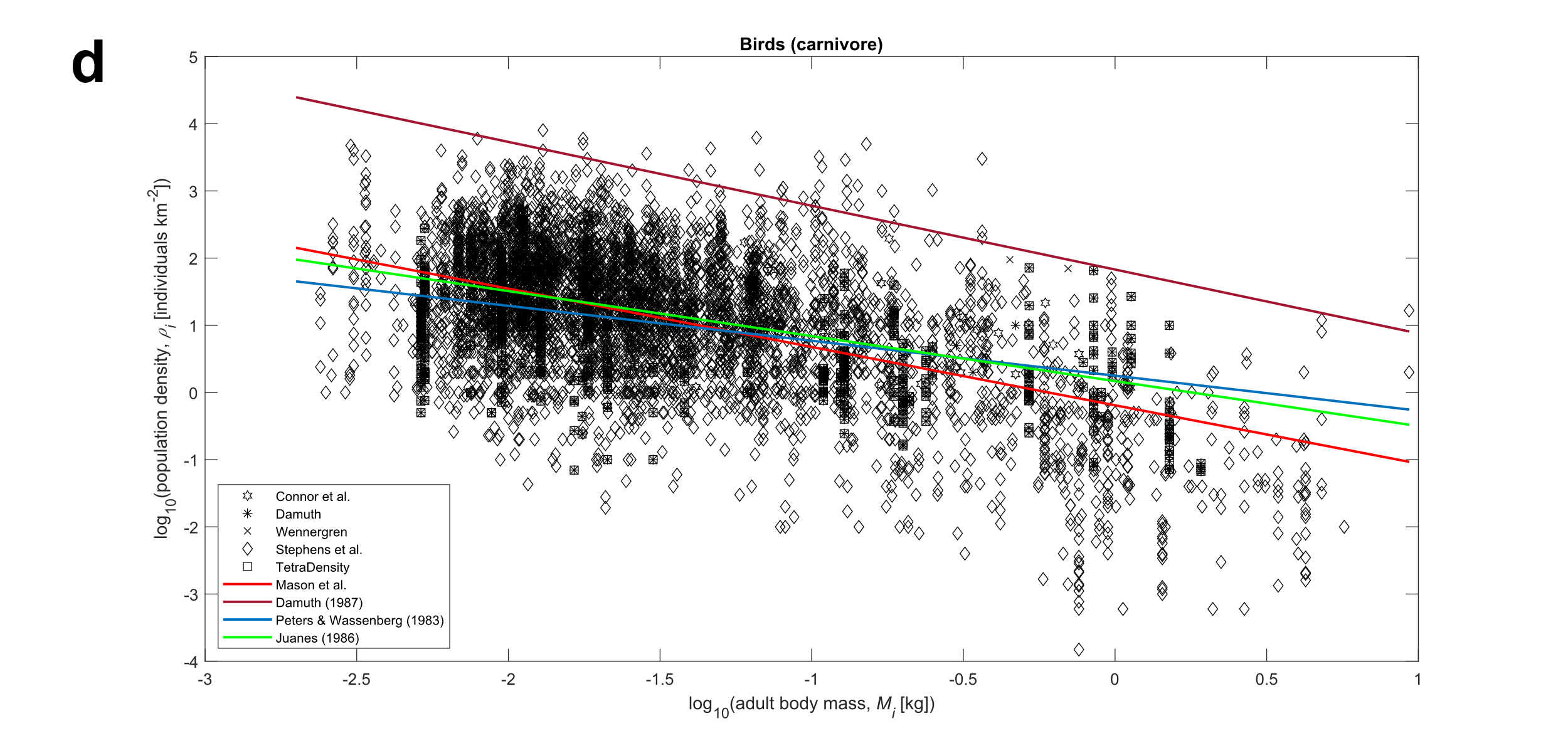


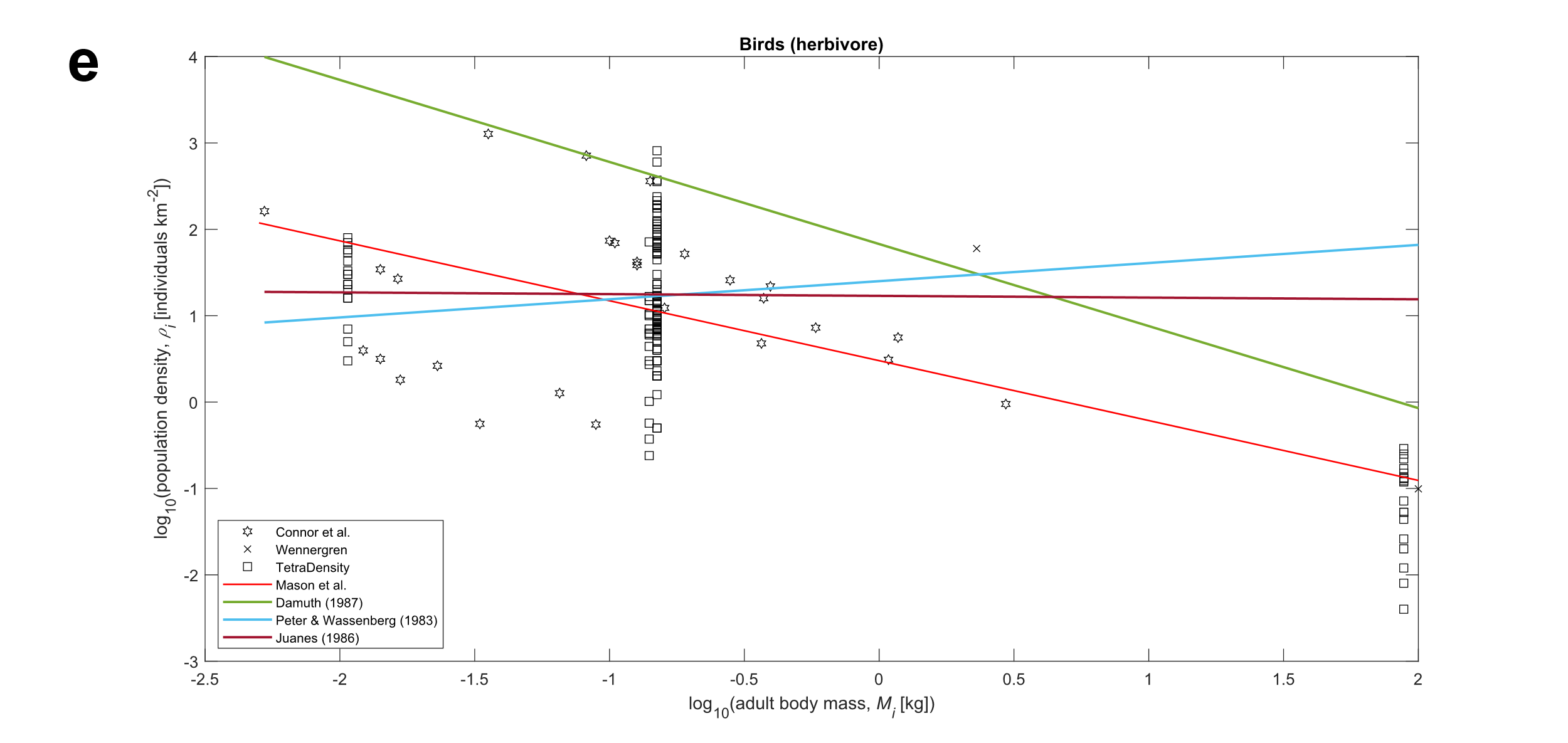


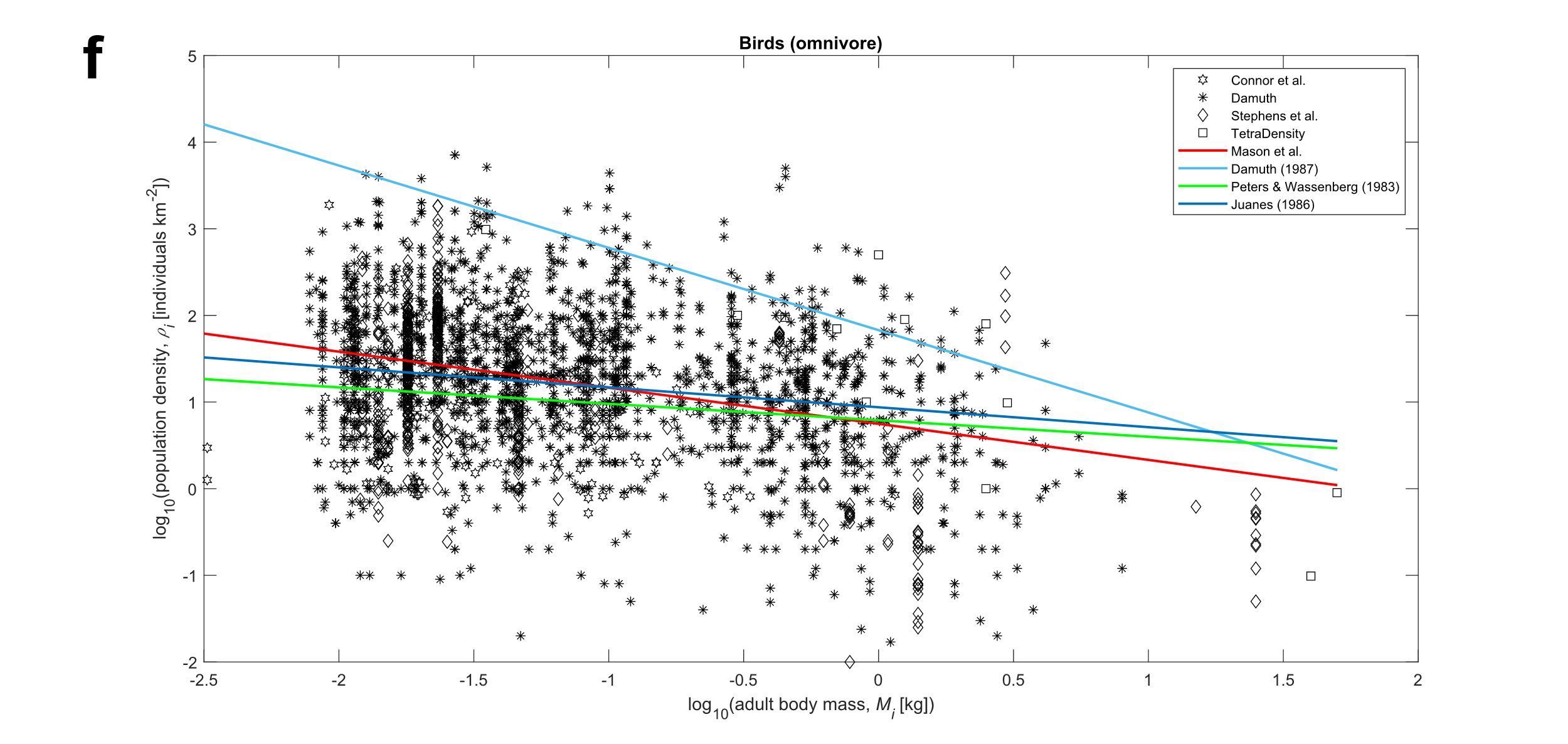


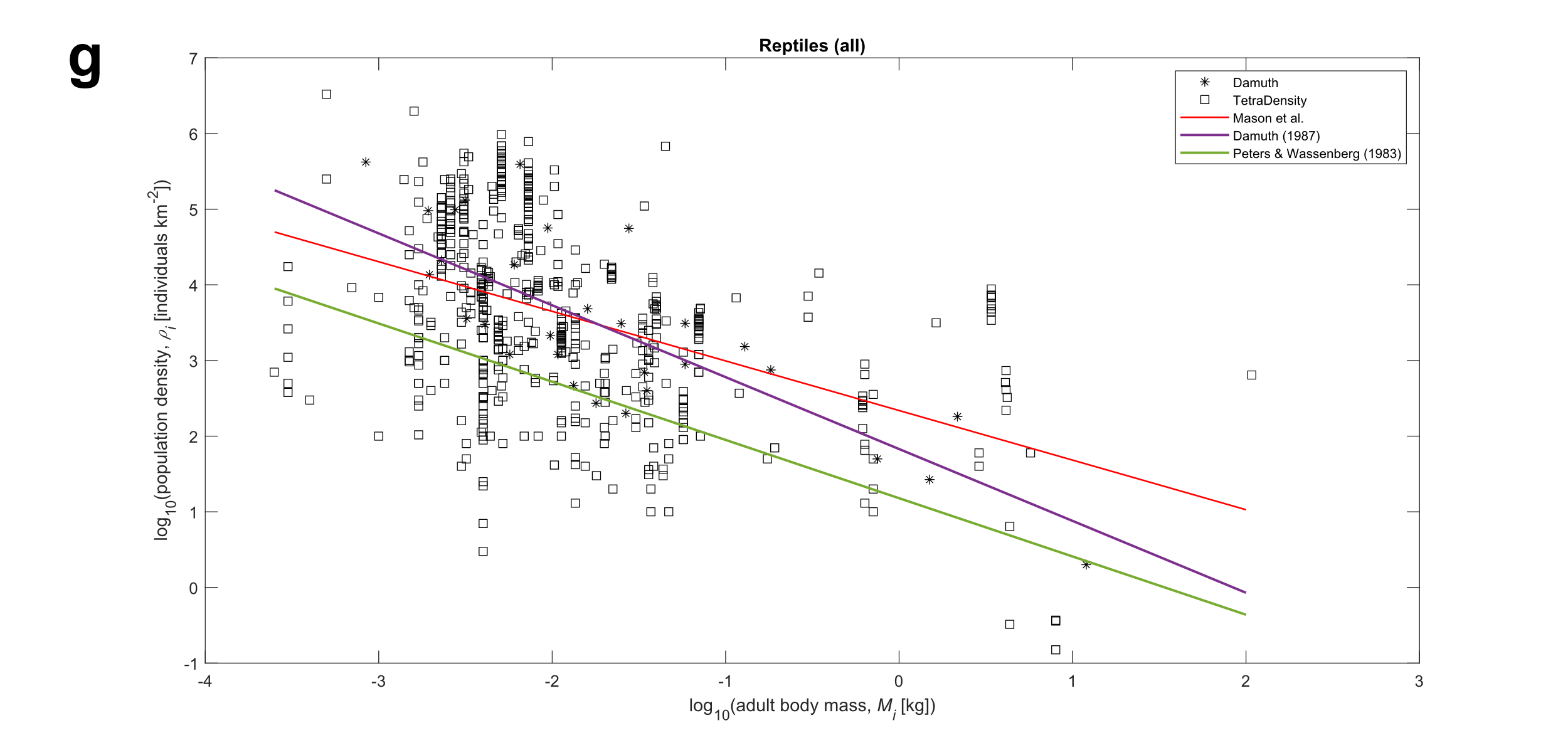


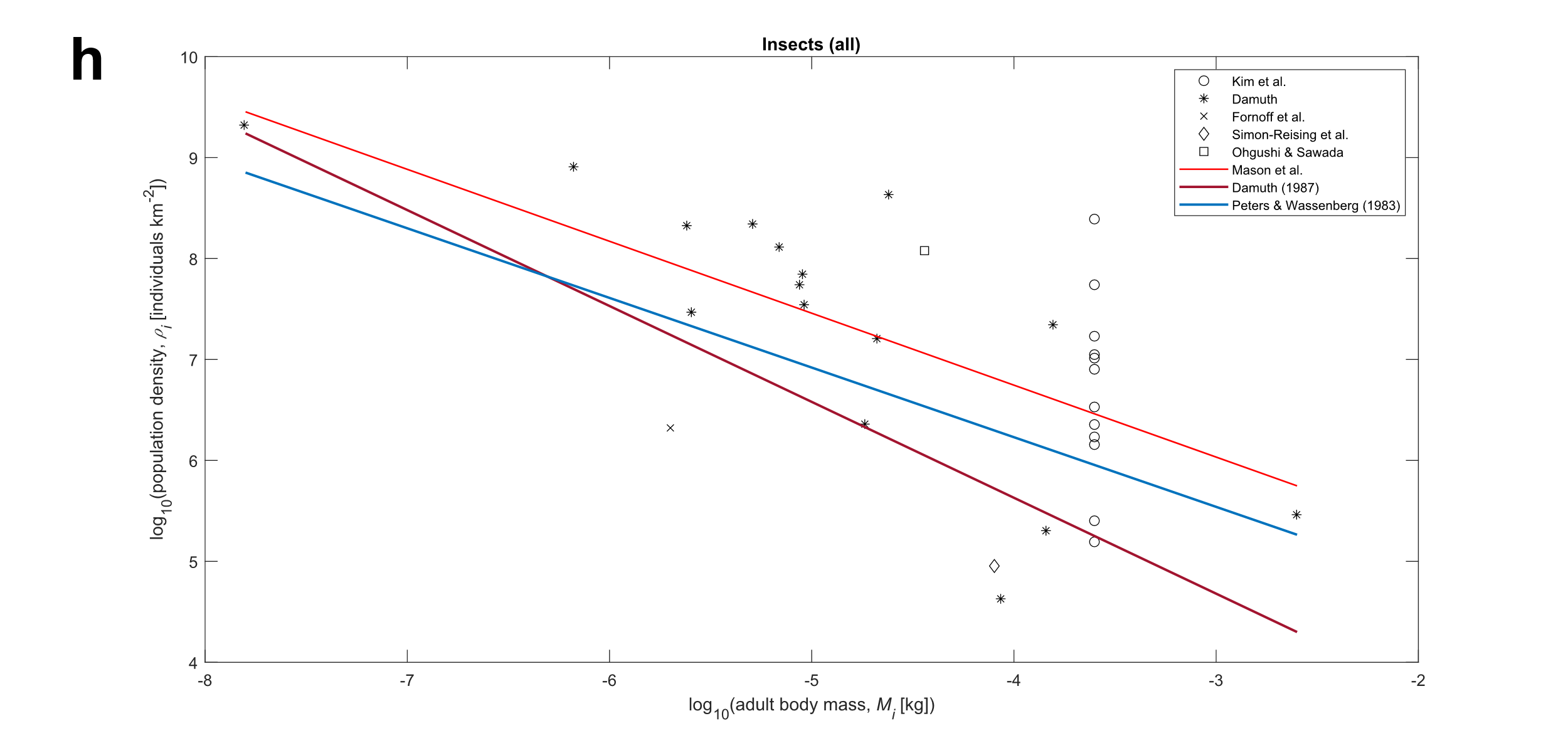


**Figure A2.** The fit of our proposed allometric equations for land area use (red line, foreground; see Table 1, Main Text) alongside that of other prominent precedents in the literature, for mammals (**a**, carnivores; **b**, omnivores; **c**, herbivores); birds (**d**, carnivores; **e**, omnivores; **f** herbivores); **g**, reptiles; and **h**, insects. The sources of the data (markers, background) used are shown in the legends. In each case, the full dataset is available in the Supplementary Data document.

In Figure A2c, the allometric equations reported by Damuth (1987) produce results that are an order of magnitude higher relative to the other results. In Figure A2d, a greater discrepancy is observed; the allometric equations reported by Damuth (1987) produce results that are 2 orders of magnitude higher relative to the other results. It is likely that this difference is the consequence of using a dataset that is much smaller than those used by the other authors referenced in Figure A2, and substantially smaller than that which is used to produce the allometric equations for land area use presented in Table 1 (Main Text).

There are significant differences between reported allometric equations for birds. Notably, the equations reported by Juanes (1976) and Peters & Wassenberg (1983) possess positive slopes and do not fit the herbivore data (Juanes, 1986; Peters & Wassenberg, 1983). This is contrary to the general trend across all the biological classes, that population density decreases with increasing body size. Additionally, the allometric equation reported by Damuth (1987) apparently over-estimates population density by 1-2 orders of magnitude relative to the others. This may be because Damuth (1987) uses a single equation to describe all non-mammalian terrestrial vertebrates (i.e., birds and reptiles in this scope) and uses a relatively small dataset for non-mammal species, both of which reduce equation accuracy. The limitation of Damuth’s small datasets is made more apparent as our expanded dataset contains a considerable quantity of mammal and bird data that was not used in previous studies. In contrast, our expanded dataset contains relatively less additional data for reptiles and insects compared to Damuth (1987). Hence the allometric equations proposed by Damuth (1987) for reptiles and insects are comparable to those we have developed and present in Table 1 (Main Text).

We note that the correlation between body mass and population density across insect species is relatively poor (Blackburn, Harvey & Pagel, 2016; Morse, Stork & Lawton, 2008), owing to a lack of reported population density data for many insect species. Until such data is collected, we are unable to predict the land area use of insects as accurately as the land area use of mammals, birds, and reptiles. However, it is important that insects are not omitted when considering the natural capital demand of ecosystems. Insects perform critical roles in ecosystem functionality and warrant the same level of consideration as the other biological classes. By including insects in our proposed set of ecosystem well-being needs, we set a precedent; we encourage others to describe the natural capital requirements of insects alongside other, better-studied biological classes. Moreover, we encourage further data collection to describe the land area use of insects more accurately than we can at this stage.
